# What a difference a methylene makes: replacing Glu with Asp or Aad in the Lys-urea-Glu pharmacophore of PSMA-targeting radioligands to reduce kidney and salivary gland uptake

**DOI:** 10.7150/thno.76571

**Published:** 2022-08-21

**Authors:** Hsiou-Ting Kuo, Kuo-Shyan Lin, Zhengxing Zhang, Chengcheng Zhang, Helen Merkens, Ruiyan Tan, Aron Roxin, Carlos F. Uribe, François Bénard

**Affiliations:** 1 Department of Molecular Oncology, BC Cancer, Vancouver, BC V5Z1L3, Canada.; 2 Department of Functional Imaging, BC Cancer, Vancouver, BC V5Z4E6, Canada.; 3 Department of Radiology, University of British Columbia, Vancouver, BC V5Z1M9, Canada.

**Keywords:** Prostate-specific membrane antigen, Salivary gland, Off-target uptake, Targeted radioligand therapy, Tumor-to-kidney absorbed dose ratio

## Abstract

The aim of this study was to investigate the effect of replacing Glu in the Lys-urea-Glu PSMA-targeting pharmacophore of [^68^Ga]Ga-HTK03041 with a close analog on the uptake of kidneys, salivary glands and PSMA-expressing tumor xenografts.

**Methods:** HTK03161, HTK03149 and HTK03189A/B were obtained by replacing Glu in HTK03041 with Asp, Aad (L-2-aminoadipic acid) and Api (2-aminopimelic acid), respectively. PSMA binding affinities were measured by competition binding assays. PET imaging and biodistribution studies of ^68^Ga-labeled ligands were performed in LNCaP tumor-bearing mice. The best candidate HTK03149 was selected and radiolabeled with ^177^Lu, and SPECT imaging and biodistribution studies were performed in LNCaP tumor-bearing mice. Radiation dosimetry calculation was conducted using the OLINDA software. Radioligand therapy study was performed in LNCaP tumor-bearing mice treated with [^177^Lu]Lu-HTK03149 (9.3-148 MBq), [^177^Lu]Lu-PSMA-617 (37 MBq) or ^nat^Lu-HTK03149 (500 pmol).

**Results:** PSMA binding affinities (K_i_) of Ga-HTK03161, Ga-HTK03149, Ga-HTK03189A and Lu-HTK03149 were 3.88±0.66, 6.99±0.80, 550±35.7 and 1.57±0.42 nM, respectively. PET imaging showed that all ^68^Ga-labeled HTK03161, HTK03149 and HTK03189A/B were excreted mainly via the renal pathway and had minimal uptake in all organs/tissues including kidneys and salivary glands. Tumor xenografts were clearly visualized in PET images of [^68^Ga]Ga-HTK03161 and [^68^Ga]Ga-HTK03149 but were barely visualized using [^68^Ga]Ga-HTK03189A/B. Tumor uptake values for [^68^Ga]Ga-HTK03161, [^68^Ga]Ga-HTK03149, [^68^Ga]Ga-HTK0189A and [^68^Ga]Ga-HTK03189B were 12.7±1.91, 19.1±6.37, 2.10±0.28 and 0.67±0.15 %IA/g, respectively at 1h post-injection, and their average kidney and salivary gland uptake values were 2.13-4.41 and 0.20-0.23 %IA/g, respectively. Longitudinal SPECT imaging studies showed that [^177^Lu]Lu-HTK03149 was excreted mainly through the renal pathway with high uptake in LNCaP tumors and minimal uptake in all normal organs/tissues. The tumor uptake of [^177^Lu]Lu-HTK03149 peaked at 4h post-injection (20.9±2.99 %IA/g) and the uptake was sustained over time. Compared to [^177^Lu]Lu-PSMA-617, [^177^Lu]Lu-HTK03149 had 145% increase in tumor absorbed dose but 70% less in kidney absorbed dose, leading to an 7.1-fold increase in tumor-to-kidney absorbed dose ratio. Radioligand therapy studies showed that only half of the [^177^Lu]Lu-PSMA-617 injected dosage was needed for [^177^Lu]Lu-HTK03149 to achieve the same median survival.

**Conclusion:** Replacing Glu in the PSMA-targeting Lys-urea-Glu pharmacophore of [^68^Ga]Ga-HTK03041 with Asp and Aad generates [^68^Ga]Ga-HTK03161 and [^68^Ga]Ga-HTK03149, respectively, and the new derivatives retain high uptake in LNCaP tumors and have minimal uptake in normal organs/tissues including kidneys and salivary glands. [^177^Lu]Lu-HTK03149 also retain high uptake in LNCaP tumors and has minimal uptake in normal organs/tissues, and is, therefore, promising for clinical translation to treat prostate cancer.

## Introduction

Prostate-specific membrane antigen (PSMA), also known as glutamate carboxypeptidase II, N-acetyl-L-aspartyl-L-glutamate peptidase I and folate hydrolase 1, is a transmembrane enzyme that is highly expressed on prostate cancer cells and in tumor-associated neovasculature of other cancers 1. Various PSMA-targeting radioligands have been developed for the detection and radioligand therapy of prostate cancer. Among them, [^18^F]DCFPyL and [^68^Ga]Ga-PSMA-11 have been approved by the US FDA for prostate cancer imaging with positron emission tomography (PET) 2-3. Radiotherapeutic agents such as [^177^Lu]Lu-PSMA-617, [^177^Lu]Lu-PSMA-I&T, [^225^Ac]Ac-PSMA-617, and [^225^Ac]Ac-PSMA-I&T are used in the clinic to treat metastatic castration-resistant prostate cancer, and [^177^Lu]Lu-PSMA-617 has also been approved by the US FDA 4-7.

Most of the reported PSMA-targeting radioligands including [^18^F]DCFPyL, [^68^Ga]Ga-PSMA-11, [^177^Lu]Lu-PSMA-617, [^177^Lu]Lu-PSMA-I&T, [^225^Ac]Ac-PSMA-617, and [^225^Ac]Ac-PSMA-I&T are derived from the Lys-urea-Glu pharmacophore, and all of them show significant uptake in kidneys and salivary glands. The high kidney uptake of PSMA-targeting ligands reduces the sensitivity for detecting prostate cancer lesions adjacent to kidneys due to the signal extinction artifact 8. In addition, the high kidney and salivary gland uptake of PSMA-targeting radiotherapeutic agents can cause xerostomia and impaired renal function especially when ^225^Ac-labeled therapeutics such as [^225^Ac]Ac-PSMA-617 and [^225^Ac]Ac-PSMA-I&T are used 7, 9.

To minimize toxicity, various strategies have been investigated to reduce kidney and salivary gland uptake of PSMA-targeting radioligands. The attempts of using external cooling with icepacks on cheeks did not significantly reduce salivary gland uptake 10. Baum et al. reported that 45 days after the injection of botulinum toxin A (80 units) into the right parotid gland of a prostate cancer patient, a [^68^Ga]Ga-PSMA-11 scan showed an up to 64% reduction in SUVmean of the treated parotid gland as compared with baseline 11, but no follow-up studies were reported. Treatment with a small amount of a potent PSMA inhibitor, such as PSMA-11, DCFPyL or PMPA, has been shown to reduce kidney/salivary gland uptake but not tumor uptake of PSMA-targeting radioligands in animal models 12-14. However, implementing this strategy in the clinic is challenging as different treatment dosages may be needed for patients with various tumor burdens and the concerns of toxicity and reduced tumor uptake when a large dosage is administrated.

**Figure 1:**
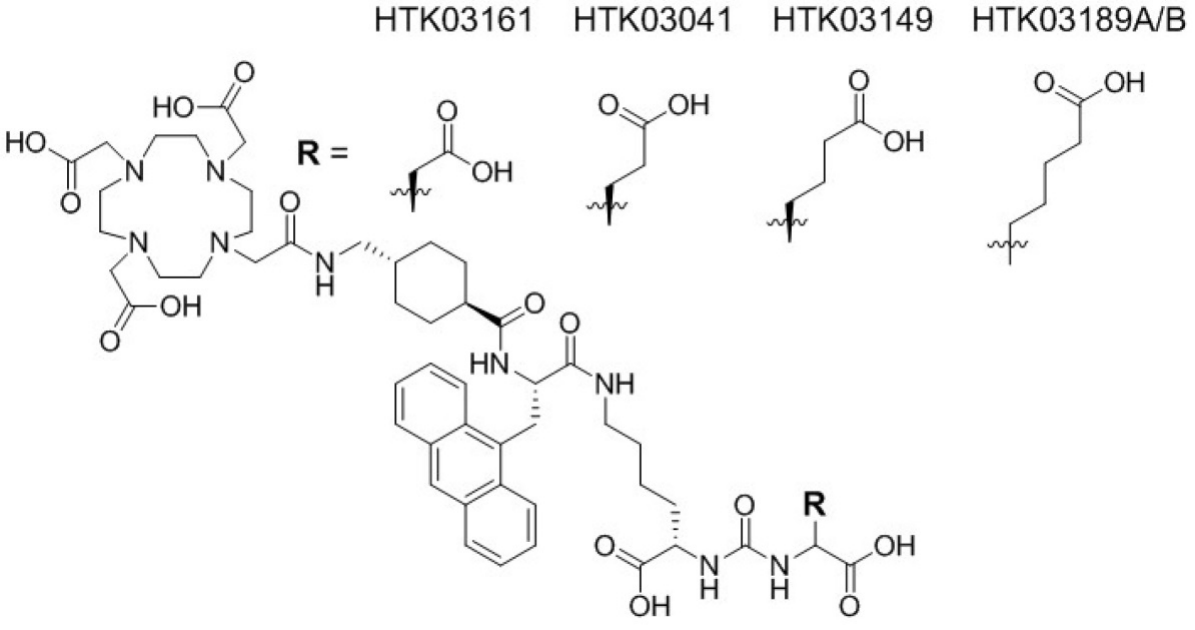
Chemical structures of HTK03161, HTK03041, HTK03149 and HTK03189A/B.

Recently we observed in a mouse model that pretreatment with up to 657 mg/kg of monosodium glutamate, a weak PSMA inhibitor (K_i_ = 0.90 µM), reduced uptake of [^68^Ga]Ga-PSMA-11 in salivary glands and kidneys but had no effect on tumor uptake 15. This suggests that some of the kidney and salivary gland uptake of [^68^Ga]Ga-PSMA-11 might not be PSMA-mediated, and moreover might be caused by the Glu moiety in the Lys-urea-Glu pharmacophore. Therefore, for this study, we investigated if the high uptake of the previously reported [^68^Ga]Ga-HTK03041 in mouse kidneys (170±26.4 %IA/g, 1h post-injection) and salivary glands (4.99±0.88 %IA/g, 1h post-injection, unreported data) can be reduced by replacing its Glu moiety in the Lys-urea-Glu pharmacophore with close analogs 16. Specifically, we replaced Glu in [^68^Ga]Ga-HTK03041 with Asp, Aad (2-aminoadipic acid) or Api (2-aminopimelic acid) to generate [^68^Ga]Ga-HTK03161, [^68^Ga]Ga-HTK03149 and [^68^Ga]Ga-HTK03189, respectively (Figure 1). We conducted *in vitro* competition binding assays, PET imaging and biodistribution studies in mice to investigate the effects of the replacement on PSMA binding affinity and uptake in kidneys, salivary glands and PSMA-expressing tumor xenografts. The best candidate HTK03149 was identified and radiolabeled with ^177^Lu, and SPECT imaging, biodistribution and radioligand therapy studies were conducted in tumor-bearing mice to evaluate the potential of [^177^Lu]Lu-HTK03149 for treating prostate cancer.

## Materials and Methods

### Cell culture

The LNCaP cells obtained from ATCC (via Cedarlane, Burlington, Canada) were cultured in RPMI 1640 medium supplemented with 10% FBS, penicillin (100 U/mL) and streptomycin (100 μg/mL) at 37 °C in a Panasonic Healthcare (Tokyo, Japan) MCO-19AIC humidified incubator containing 5% CO_2_. The cells were confirmed to be pathogen-free by the IMPACT Rodent Pathogen Test (IDEXX BioAnalytics). Cells grown to 80-90% confluence were then washed with sterile phosphate-buffered saline (PBS, pH 7.4) and collected after trypsinization. The cell concentration was counted in triplicate using a hemocytometer and a manual laboratory counter.

### *In vitro* competition binding assays

The binding assays were conducted following previously published procedures using LNCaP cells and [^18^F]DCFPyL as the radioligand 17-19. Briefly, LNCaP cells (400,000/well) were plated onto a 24-well poly-D-lysine coated plate for 48 h. Growth media was removed and replaced with HEPES buffered saline (50 mM HEPES, pH 7.5, 0.9% sodium chloride) and the cells were incubated for 1 h at 37 °C. [^18^F]DCFPyL (0.1 nM) was added to each well (in triplicate) containing various concentrations (0.5 mM - 0.05 nM) of tested nonradioactive Ga-complexed compounds. Non-specific binding was determined in the presence of 10 µM non-radiolabeled DCFPyL. The assay mixtures were further incubated for 1 h at 37 °C with gentle agitation. Then, the buffer and radioligand were removed, and cells were washed twice with cold HEPES buffered saline. To harvest the cells, 400 µL of 0.25 % trypsin solution was added to each well. Radioactivity was measured on a PerkinElmer (Waltham, MA) Wizard2 2480 automatic gamma counter. Data analyses of K_i_ were performed using the nonlinear regression algorithm of GraphPad (San Diego, CA) Prism 7 software.

### Biodistribution, imaging and radioligand therapy studies

Imaging and biodistribution experiments were performed following previously published procedures using male NOD-*scid* IL2Rg^null^ (NSG) mice for the ^68^Ga-labeled compounds and NOD.Cg-Rag1^tm1Mom^ Il2rg^tm1Wjl^/SzJ (NRG) mice for [^177^Lu]Lu-PSMA-617 and [^177^Lu]Lu-HTK03149 16-19. The experiments were conducted according to the guidelines established by the Canadian Council on Animal Care and approved by Animal Ethics Committee of the University of British Columbia (Certificate number: A20-0113). The mice were briefly sedated by inhalation of 2% isoflurane in oxygen and subcutaneously implanted with 1×10^7^ LNCaP cells behind the left shoulder. The mice were imaged or used in biodistribution studies when the tumor grew to 5-8 mm in diameter over a period of 5-6 weeks (success rate ≈ 80%).

PET/CT imaging experiments were conducted using a Siemens (Knoxville, TN) Inveon micro PET/CT scanner. Each tumor-bearing mouse (n = 1-2) was injected with ~6-8 MBq (10-150 pmol) of ^68^Ga-labeled ligand through a lateral caudal tail vein. At 50 min post-injection, a 10-min CT scan was conducted first for localization and attenuation correction after segmentation for reconstructing the PET images, followed by a 10-min static PET imaging acquisition. PET imaging data were acquired in list mode, reconstructed using 3-dimensional ordered-subsets expectation maximization (2 iterations) followed by a fast maximum a priori algorithm (18 iterations) with CT-based attenuation correction. Images were analyzed using Inveon Research Workplace software (Siemens Healthineers).

SPECT/CT imaging experiments were conducted using the MILabs (Utrecht, The Netherlands) U-SPECT-II/CT scanner. Each tumor-bearing mouse (n = 1-2 was injected with ∼37 MBq (50-250 pmol) of ^177^Lu-labeled compound through a lateral caudal tail vein. The mice were imaged at 1, 4, 24, 72, and 120 h after injection. At each time point, a 5-min CT scan was conducted first for anatomical reference followed by 2 × 30-min static emission scans acquired in list mode.

For biodistribution studies, the mice (n = 4-8) were injected with the radioligand (2-4 MBq, 5-75 pmol) as described above. For blocking, the mice (n = 4) were co-injected with nonradioactive DCFPyL (0.5 mg). At predetermined time points, the mice were sedated by isoflurane inhalation and euthanized by CO_2_ inhalation. Blood was withdrawn by cardiac puncture, and organs/tissues of interest were collected, weighed and counted using a Perkin Elmer (Waltham, MA) Wizard2 2480 automatic gamma counter.

For radioligand therapy studies, tumor-bearing NRG mice were injected with PBS, [^177^Lu]Lu-PSMA-617 (37 MBq), ^nat^Lu-HTK03149 (500 pmol) or [^177^Lu]Lu-HTK03149 (148, 74, 37, 18.5, or 9.3 MBq) (n = 7-8 per group). Tumor size (determined by Biopticon Imager 2, Software TumorManager 3.3.2) and body weight were measured twice a week from the date of injection (Day 0) until completion of the study (Day 180). Endpoint criteria were defined as > 15% weight loss, tumor volume > 1,000 mm^3^, or active ulceration of the tumor.

## Results

### Synthesis of PSMA-targeting ligands and their binding affinities

**Figure 2:**
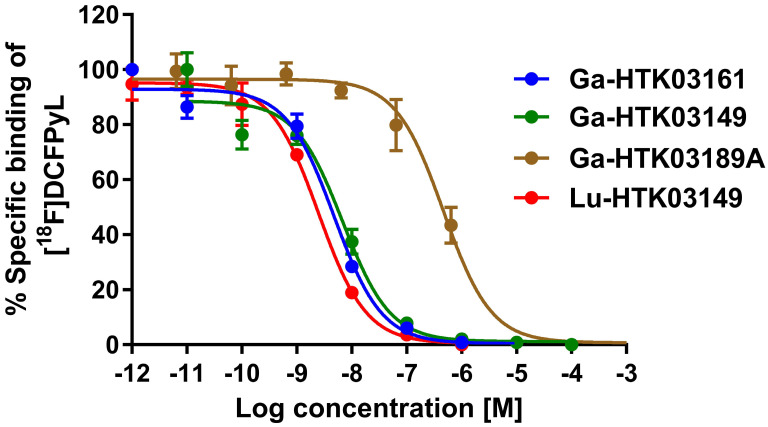
Representative displacement curves of [^18^F]DCFPyL by Ga- and Lu-complexed PSMA-targeting ligands.

Detailed information for the syntheses of DOTA-conjugated PSMA-targeting ligands, their nonradioactive Ga- and Lu-complexed standards, and their ^68^Ga- and ^177^Lu-labeled analogs is provided in the Supplemental Information (Tables S1-S3). To remove potential radiolabeled impurities and free ^68^Ga/^177^Lu, HPLC purification was performed for the preparation of all ^68^Ga- and ^177^Lu-labeled ligands. Since racemic Api was used for the synthesis of HTK03189, two isomers (HTK03189A and HTK03189B) were obtained after HPLC purification. As shown in Figure 2, Ga- and Lu-complexed PSMA-targeting ligands inhibited the binding of [^18^F]DCFPyL to LNCaP prostate cancer cells in a dose-dependent manner. The calculated K_i_ values for Ga-HTK03161, Ga-HTK03149, Ga-HTK03189A and Lu-HTK03149 are 3.88±0.66, 6.99±0.80, 550±35.7 and 1.57±0.42 nM (n = 3), respectively. The K_i_ value of Lu-PSMA-617 was reported previously to be 0.24±0.06 nM using the same assay conditions 18.

### PET imaging and *ex vivo* biodistribution of ^68^Ga-labeled ligands

**Figure 3:**
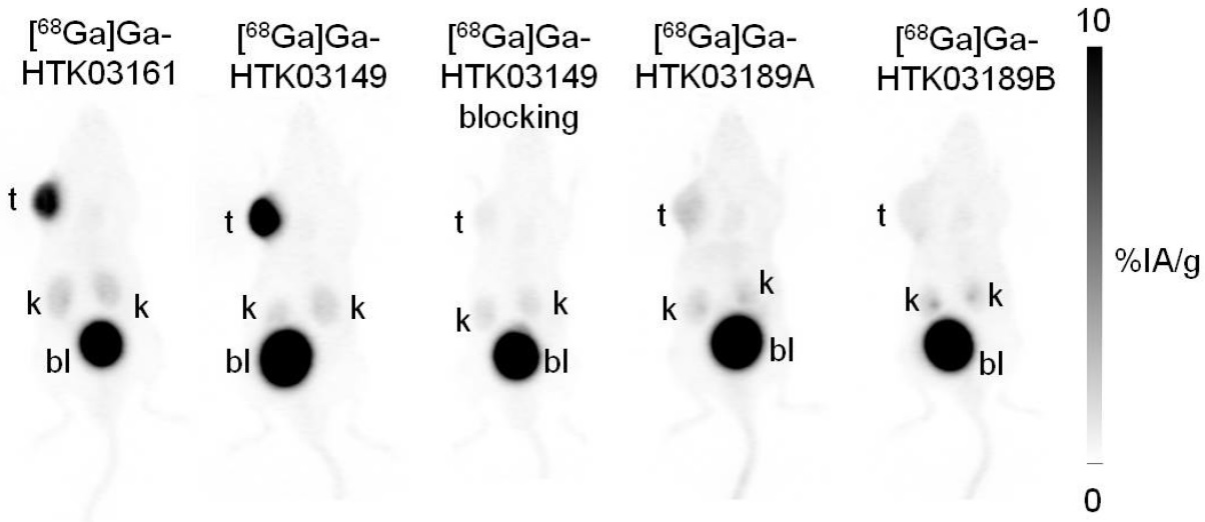
Representative maximum intensity projection PET images of ^68^Ga-labeled PSMA-targeting ligands acquired at 1h post-injection from LNCaP tumor-bearing mice.

PET imaging studies showed that all of the evaluated ^68^Ga-labeled PSMA-targeting ligands were excreted mainly via the renal pathway and had minimal uptake in normal organs/tissues including salivary glands and kidneys except urinary bladders (Figure 3). [^68^Ga]Ga-HTK03149, with the Lys-urea-Aad pharmacophore, had the highest uptake in PSMA-expressing LNCaP tumor xenografts, followed by the Lys-urea-Asp derivative, [^68^Ga]Ga-HTK03161. A lower tumor uptake was observed for the two Lys-urea-Api derivatives, with [^68^Ga]Ga-HTK03189A having a slightly higher tumor uptake than [^68^Ga]Ga-HTK03189B. Co-injection of DCFPyL (0.5 mg) reduced the tumor uptake of [^68^Ga]Ga-HTK03149 to close to the background level.

**Figure 4:**
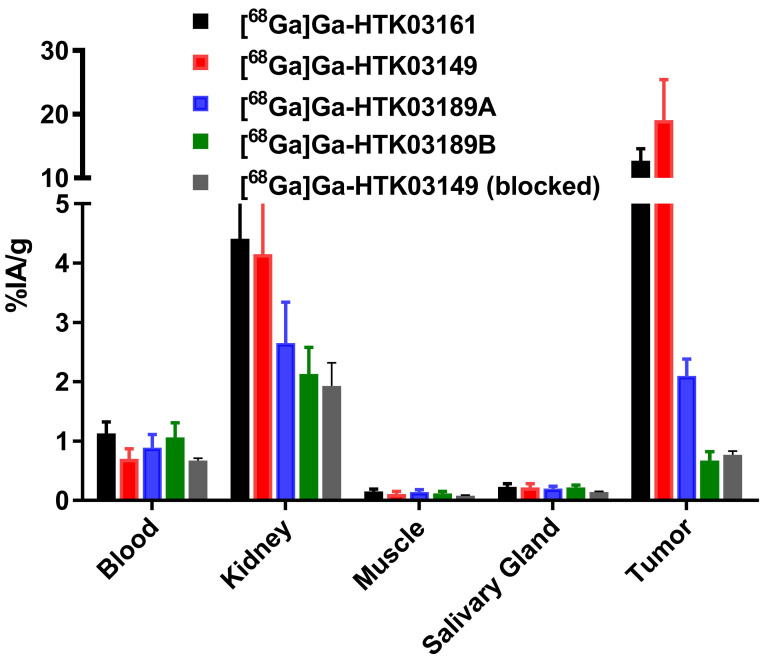
Uptake of ^68^Ga-labeled HTK03161, HTK03149, HTK03189A and HTK03189B in tumor and representative organs/tissues of LNCaP tumor-bearing mice collected at 1h post-injection.

The results of *ex vivo* biodistribution studies, conducted at 1h post-injection, for the ^68^Ga-labeled PSMA-targeting ligands are consistent with the observations from their PET images (Figure 4 and Table S4). The tumor uptake values of [^68^Ga]Ga-HTK03161, [^68^Ga]Ga-HTK03149, [^68^Ga]Ga-HTK03189A and [^68^Ga]Ga-HTK03189B were 12.7±1.91, 19.1±6.37, 2.10±0.28 and 0.67±0.15 %IA/g, respectively. The average kidney uptake values of these ^68^Ga-labeled ligands were only 2.13-4.41 %IA/g, and the average uptake values for all of the other collected organs/tissues were ~1 %IA/g or less, including only 0.20-0.23 %IA/g for salivary glands. With the highest tumor uptake, [^68^Ga]Ga-HTK03149 showed the highest tumor-to-background contrast ratios: tumor-to-blood, 29.5±15.8; tumor-to-muscle, 185±79.6; tumor-to-kidney, 5.44±3.88; tumor-to-salivary gland, 97.3±59.2. Co-injection of DCFPyL (0.5 mg) reduced the average uptake of [^68^Ga]Ga-HTK03149 in LNCaP tumor xenografts, kidneys and salivary glands by 96, 53 and 36%, respectively.

### SPECT imaging and *ex vivo* biodistribution of [^177^Lu]Lu-HTK03149

**Figure 5:**
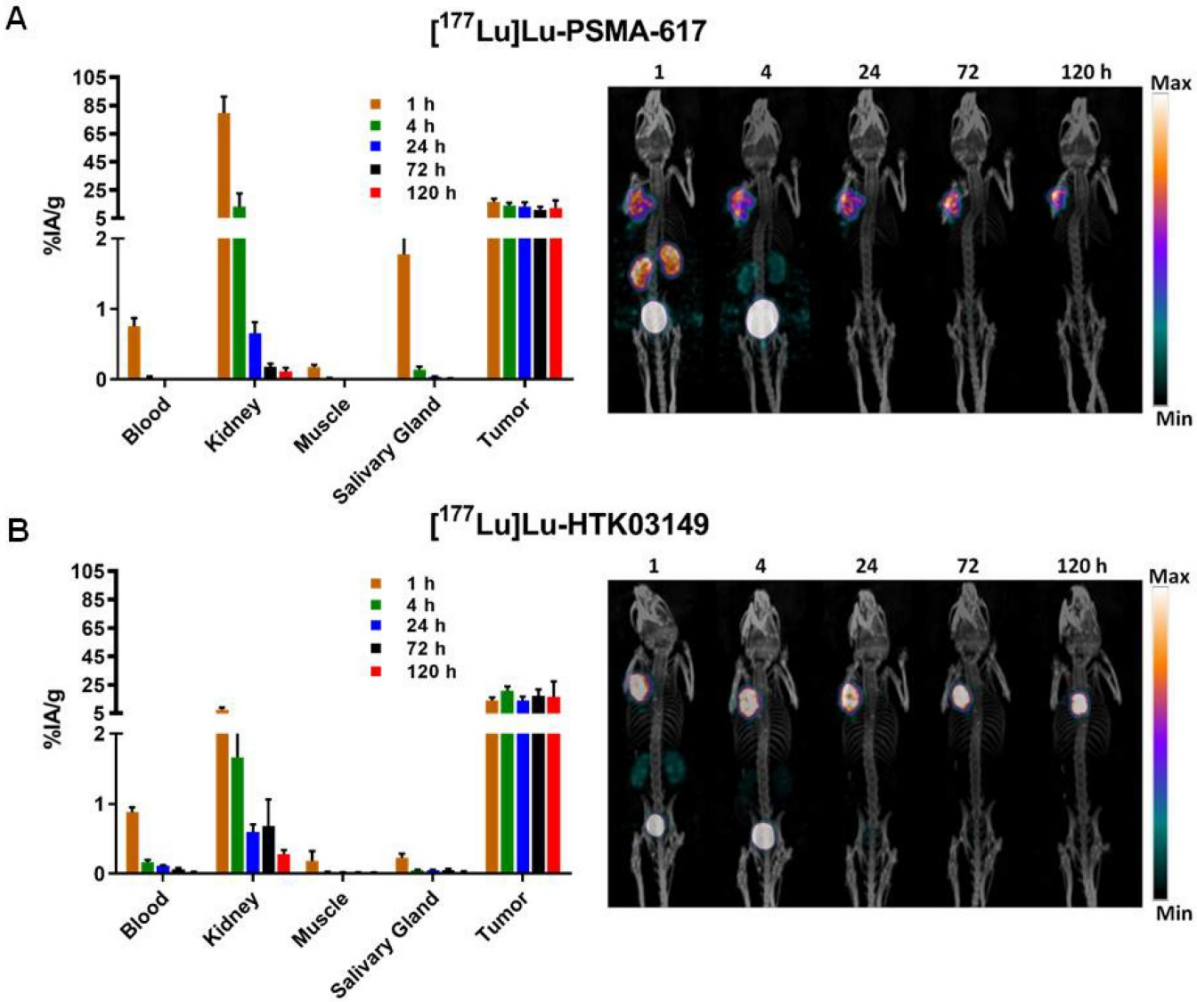
Longitudinal SPECT/CT images and *ex vivo* biodistribution of (A) [^177^Lu]Lu-PSMA-617 and (B) [^177^Lu]Lu-HTK03149 in mice bearing LNCaP tumor xenografts.

The longitudinal SPECT/CT images of [^177^Lu]Lu-PSMA-617 and [^177^Lu]Lu-HTK03149 are shown in Figure 5. Both [^177^Lu]Lu-PSMA-617 and [^177^Lu]Lu-HTK03149 were excreted mainly via the renal pathway. However, [^177^Lu]Lu-PSMA-617 exhibited a much higher uptake level in kidneys especially at the earlier time points. High uptake of [^177^Lu]Lu-PSMA-617 and [^177^Lu]Lu-HTK03149 in LNCaP tumors was also observed and the uptake level was relatively sustained over time. The tumors shrank from Day 1 to Day 5, indicating a radiotherapeutic effect from the ~37 MBq of injected [^177^Lu]Lu-PSMA-617 and [^177^Lu]Lu-HTK03149.

The results of *ex vivo* biodistribution of [^177^Lu]Lu-PSMA-617 and [^177^Lu]Lu-HTK03149 in LNCaP tumor-bearing mice at various time points after injection are shown in Figure 5 and Tables S5-S6. The tumor uptake of [^177^Lu]Lu-PSMA-617 peaked at 1h post-injection (16.5±2.46 %IA/g), and the tumor uptake level was reduced slowly over time (11.0±2.12 %IA/g at 72h post-injection). On the other hand, the tumor uptake of [^177^Lu]Lu-HTK03149 peaked at 4h post-injection (20.9±2.99 %IA/g), and the tumor uptake level was relatively sustained over time (17.1±4.70 %IA/g at 72h post-injection). [^177^Lu]Lu-PSMA-617 showed much higher kidney uptake values at the earlier time points (79.7±11.7 %IA/g at 1h post-injection and 13.1±9.60 %IA/g at 4h post-injection), while the kidney uptake of [^177^Lu]Lu-HTK03149 was only 7.67±1.35 %IA/g at 1h post-injection, which further reduced to 1.67±0.38 %IA/g at 4h post-injection. Compared with [^177^Lu]Lu-HTK03149, [^177^Lu]Lu-PSMA-617 also showed much higher salivary gland uptake values at the earlier time points (0.23±0.06 vs 1.78±0.38 %IA/g at 1h post-injection and 0.05±0.01 vs 0.14±0.04 %IA/g at 4h post-injection).

### Dosimetry and radioligand therapy

Based on the *ex vivo* biodistribution data, the absorbed doses of [^177^Lu]Lu-HTK03149 in LNCaP tumor and major mouse organs/tissues were calculated using the OLINDA software and were compared with those of [^177^Lu]Lu-PSMA-617. Regardless of simulated sphere (tumor) sizes, [^177^Lu]Lu-HTK03149 delivered 145% higher absorbed radiation dose than [^177^Lu]Lu-PSMA-617 to the LNCaP tumors (Table S7).

**Figure 6:**
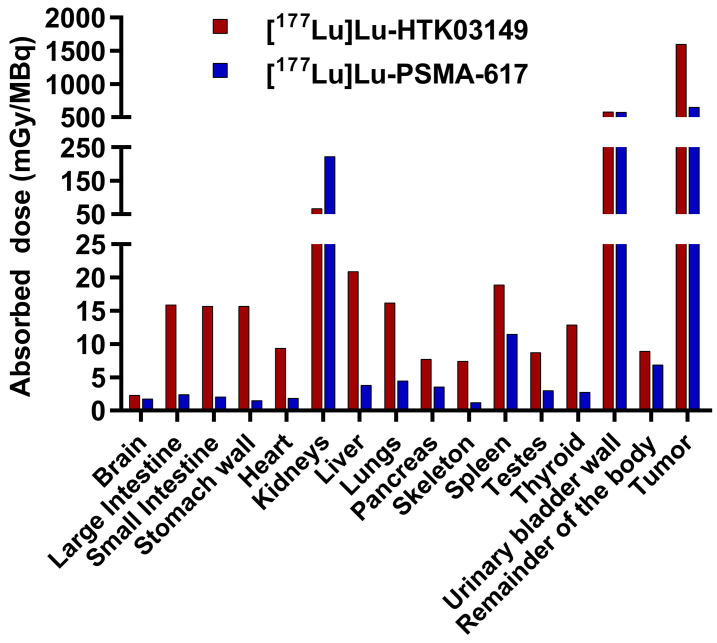
Absorbed radiation doses (mGy/MBq) delivered by [^177^Lu]Lu-HTK03149 and [^177^Lu]Lu-PSMA-617 to LNCaP tumor xenografts (0.5 g unit density sphere) and major organs/tissues of a 25-g mouse, calculated using the OLINDA software.

The input kinetics of the mouse source organs calculated from the data fit (MBq-h/MBq), and the doses to the target organs (mGy/MBq) are presented in Figure 6 and Table S8. The absorbed dose ratios of [^177^Lu]Lu-HTK03149-to-[^177^Lu]Lu-PSMA-617 were lower for kidneys (0.30), were higher (1.64 - 10.4) for intestines, stomach, heart, liver, lungs, pancreas, skeleton, spleen, testes and thyroid, and were comparable (1.31-1.01) for brain, urinary bladder wall and the remainder of the body.

**Figure 7:**
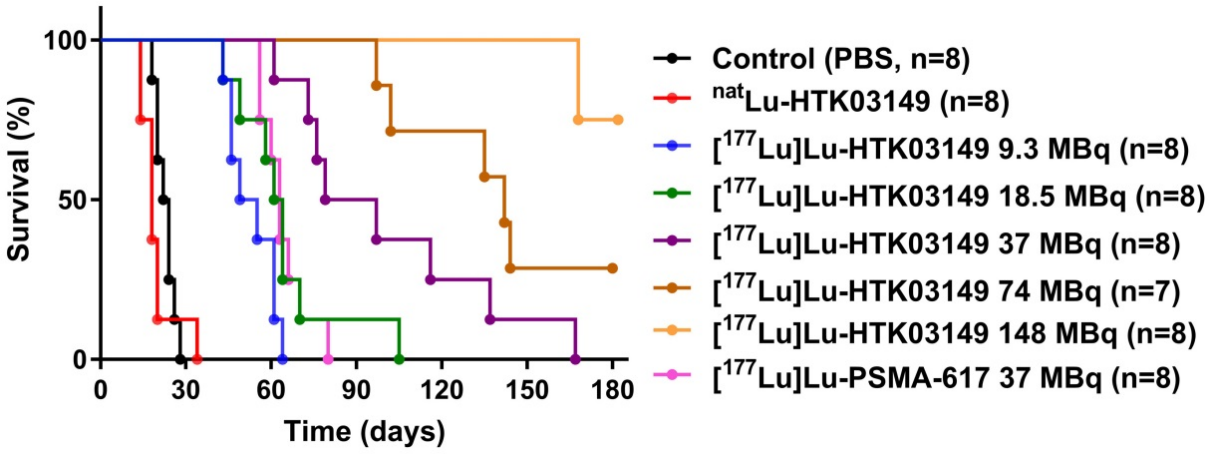
Overall survival for LNCaP tumor-bearing mice (n = 7-8 per group) injected with PBS, ^nat^Lu-HTK03149, [^177^Lu]Lu-PSMA-617 (37 MBq) or [^177^Lu]Lu-HTK03149 (9.3 - 148 MBq).

The radioligand therapy study of [^177^Lu]Lu-HTK03149 was conducted in LNCaP tumor-bearing mice using PBS, ^nat^Lu-HTK03149 and [^177^Lu]Lu-PSMA-617 as controls, and the results are provided in Figure 7 and Table 1. The mice injected with PBS or ^nat^Lu-HTK03149 had median survivals of only 23 and 18 days, respectively. Injection of 37 MBq of [^177^Lu]Lu-PSMA-617 extended the median survival to 63 days. The median survivals for the mice injected with [^177^Lu]Lu-HTK03149 depended on the injected dosages: 52, 63, 88, 142 and >180 days for 9.3, 18.5, 37, 74 and 148 MBq, respectively. No signifant weight loss was observed for the mice in any of the treated groups (Figures S1-S8).

## Discussion

The reported preclinical data of PSMA-targeting radioligands derived from the Lys-urea-Glu pharmacophore all showed blockable specific uptake in mouse kidneys and salivary glands. However, other evidence suggests that the uptake in kidneys and salivary glands might not be entirely mediated by PSMA. This includes: (1) our reported data showing the use of monosodium glutamate to reduce uptake of [^68^Ga]Ga-PSMA-11 in mouse kidneys and salivary glands but not LNCaP tumor xenografts 15; (2) using IHC staining on human tissues, Mhawech-Fauceglia reported no PSMA expression in salivary glands and only weak PSMA expression in kidneys 20; (3) the observation that there is no significant salivary gland uptake of radiolabeled PSMA-targeting antibodies (such as ^111^In-J591) observed in clinical trials 21; and (4) by comparing the low PSMA IHC staining and a minimal [^177^Lu]Lu-PSMA-617 autoradiography signal on human submandibular glands with their high [^68^Ga]Ga-PSMA-11 uptake in PET images, Bakht and co-workers concluded that the significant accumulation of PSMA radioligands in submandibular glands is not primarily a result of PSMA-mediated uptake 22.

Replacing Glu in the widely used Lys-urea-Glu pharmacophore for the development of novel PSMA-targeting radioligands has also been attempted by others. Yang replaced Glu with (S)-2-amino-3-(carboxyformamido)propanoic acid and used various linkers between the DOTA chelator and the new PSMA-targeting pharmacophore. PSMA-targeting capability of these new derivatives was preserved but the uptake of their ^68^Ga-labeled analogs in mouse kidneys was comparable or higher than that of [^68^Ga]Ga-PSMA-617 23. Felber reported the replacement of Glu in the Lys-urea-Glu pharmacophore of Lu-PSMA-10 (**1**) with (S)-2-aminoheptanoic acid (**8**), (S)-2-amino-3-(furan-2-yl)propanoic acid (**9**), (S)-2-aminopent-4-ynoic acid (**10**) and (S)-2-amino-4-(1H-1,2,3,4-tetrazol-5-yl)butanoic acid (**11**). However, compared to their Glu analog Lu-PSMA-10 (IC_50_ = 2.8 nM), the binding affinities (IC_50_) of **8**, **9**, **10** and **11** were >2000, >440, 138 and 16.4 nM, respectively. Biodistribution studies also showed that compared to [^177^Lu]Lu-PSMA-10, [^177^Lu]Lu-**11** had a lower kidney uptake (173 vs 33.2 %IA/g, 1h post-injection) but also a lower uptake in LNCaP tumor xenografts (12.2 vs 3.4 %IA/g) 24.

In contrast to the attempts by others, we replaced Glu in the previously reported [^68^Ga]Ga-HTK03041 with the more closely related analogs, Asp, Aad or Api, which have a side chain one methylene shorter, one methylene longer, or two methylenes longer than Glu, respectively. *In vitro* competition binding assays showed that the Asp (Ga-HTK03161) and Aad (Ga-HTK03149) derivatives retained good PSMA binding affinity, indicating that adding or deleting one methylene on the Glu side chain does not affect PSMA binding affinity. However, adding two methylenes to the Glu side chain significantly reduced its PSMA binding capability, as the K_i_ value of Ga-HTK03189A was 550 nM. The PET imaging and biodistribution data of these ^68^Ga-labeled PSMA-targeting ligands were consistent with their binding affinities, as only the Asp ([^68^Ga]Ga-HTK03161) and Aad ([^68^Ga]Ga-HTK03149) derivatives showed good uptake in PSMA-expressing LNCaP tumor xenografts. The LNCaP tumor uptake values of [^68^Ga]Ga-HTK03189A and [^68^Ga]Ga-HTK03189B were 2.10±0.28 and 0.67±0.15 %IA/g, respectively. Two isomers of HTK03189 were obtained after HPLC purification due to the use of racemic Api for the synthesis of HTK03189. The configurations (i.e. D-Api vs L-Api) of these two HTK03189 isomers (A and B) were not confirmed. However, based on the fact that [^68^Ga]Ga-HTK03189A had ~2-fold higher tumor uptake than [^68^Ga]Ga-HTK03189B, it is more likely that [^68^Ga]Ga-HTK03189A and [^68^Ga]Ga-HTK03189B contain L-Api and D-Api, respectively.

The most intriguing observations were the extremely low uptake of [^68^Ga]Ga-HTK03161 and [^68^Ga]Ga-HTK03149 in mouse kidneys (4.41±1.26 and 4.15±1.46 %IA/g, respectively) and salivary glands (0.23±0.05 and 0.22±0.06, respectively) at 1h post-injection as compared to those of previously reported [^68^Ga]Ga-HTK03041 (kidney uptake: 170±26.4 %IA/g; salivary gland uptake: 4.99±0.88 %IA/g) 16. This is unlikely to be caused by the injected mass of PSMA-targeting ligands. This is because we used HPLC purification to remove unlabeled precursors to ensure high molar activity of the radioligands, and the total injected mass for the biodistribution studies was estimated to be only up to 75 pmol. To the best of our knowledge, most of the reported small-molecule PSMA-targeting radioligands have <1.0 tumor-to-kidney uptake ratios at 1h post-injection. In addition, we have not seen a >5.0 LNCaP tumor-to-kidney uptake ratio at 1h post-injection from any reported ^68^Ga-labeled small-molecule PSMA-targeting ligands, as observed with [^68^Ga]Ga-HTK03149. Such a high tumor-to-kidney uptake ratio will not only enable the detection of metastatic prostate cancer lesions adjacent to kidneys, but may also enable the detection of primary renal cell carcinoma which expresses a high density of PSMA 25.

Compared to human salivary glands, PSMA is expressed in a much lower level in mouse salivary glands and, therefore, mice might not be a good model for evaluating salivary gland uptake of PSMA-targeting radioligands 26. However, our preclinical data, generated from the use of Lys-urea-Glu-based PSMA-targeting radioligands (including [^177^Lu]Lu-PSMA-617, Table S5), all showed <1.0 blood-to-salivary gland uptake ratios at 1h post-injection. This suggests that there is an active uptake mechanism of Lys-urea-Glu-based radioligands into mouse salivary glands. On the contrary, the blood-to-salivary gland uptake ratios of [^68^Ga]Ga-HTK03161 and [^68^Ga]Ga-HTK03149 at 1h post-injection were 5.08±0.62 and 3.21±0.64, respectively (Table S4). This suggests the uptake of [^68^Ga]Ga-HTK03161 and [^68^Ga]Ga-HTK03149 into mouse salivary glands is much slower than the Lys-urea-Glu-based PSMA-targeting radioligands. Despite the low uptake of [^68^Ga]Ga-HTK03149 in both kidneys and salivary glands, the fact that blocking with DCFPyL can further reduced its uptake in kidneys (by 53%) and salivary glands (by 36%) suggests that there is still some specific uptake of [^68^Ga]Ga-HTK03149 in these two tissues either mediated by PSMA and/or off-targets.

The most potential application of our discoveries is for radioligand therapy to minimize toxicities to kidneys and salivary glands. Therefore, the top candidate HTK03149 was labeled with ^nat^Lu/^177^Lu and evaluated by competition binding, SPECT imaging and biodistribution studies. Consistent with the observation from its ^68^Ga analog, [^177^Lu]Lu-HTK03149 showed good PSMA binding affinity and had high tumor uptake but minimal uptake in normal organs/tissues including kidneys and salivary glands. This suggests replacing the ^68^Ga label in [^68^Ga]Ga-HTK03149 with ^177^Lu does not affect its PSMA targeting capability and selective binding to PSMA over the off-targets in kidneys and salivary glands. [^177^Lu]Lu-HTK03149 delivers 145% higher absorbed dose to LNCaP tumor xenografts than [^177^Lu]Lu-PSMA-617 because of its higher peak tumor uptake (20.9±2.99 %IA/g) and sustained tumor retention. In contrast, the reported LNCaP tumor uptake of [^177^Lu]Lu-PSMA-617 peaked at 1h post-injection (16.5±2.46 %IA/g) and gradually reduced to 12.3±5.52 %IA/g at 120h post-injection. Compared to [^177^Lu]Lu-PSMA-617, the higher absorbed doses to normal organs/tissues for [^177^Lu]Lu-HTK03149 (Figure 6) is due to its longer blood residence time. This might be resulted from the enhanced interaction between plasma proteins and the 9-anthryl moiety in the lipophilic linker of [^177^Lu]Lu-HTK03149.

Radioligand therapy studies confirmed the results of dosimetry calculation, as at the half of the injected dosage (18.5 MBq), [^177^Lu]Lu-HTK03149 led to the same median survival (63 days) as 37 MBq of [^177^Lu]Lu-PSMA-617. At the same injected radioactivity (37 MBq), [^177^Lu]Lu-HTK03149 led to an extended median survival of 88 days. We raised the injected radioactivity of [^177^Lu]Lu-HTK03149 to as high as 148 MBq (Figure S8), but did not observe any weight loss for the treated mice, confirming the great safety profile of [^177^Lu]Lu-HTK03149.

We did not conduct *in vivo* stability studies for the reported ^68^Ga- and ^177^Lu-labeled PSMA-617 derivatives as they are expected to be highly stable *in vivo*. This is supported by (1) these reported radioligands are not expected to be metabolized by peptidases as there is no amide bond formed between two natural amino acids; (2) it has been shown previously that there was no radiometabolite of [^18^F]AlF-PSMA-NF, a close PSMA-617 derivative 27, observed in mouse blood, urine and kidneys 30 min post-injection; and (3) the PSMA-617 construct has been utilized by various groups for the design of albumin-binder-conjugated radiotherapeutic agents 18, 28-29, and all of them show continuously increased tumor uptake in the first 24 h post-injection, supporting the high *in vivo* stability of the PSMA-617 construct.

The reason for replacing Glu in the pharmacophore to reduce kidney and salivary gland uptake of PSMA-targeting radioligands was because of the report showing the use of monosodium glutamate to reduce uptake of [^68^Ga]Ga-PSMA-11 in kidneys and salivary glands but not LNCaP tumor xenografts 15. However, it should be noted that recent clinical studies showed that the ingestion of 12.7 g and 18.9 g of monosodium glutamate reduced the uptake of [^18^F]DCFPyL and [^68^Ga]Ga-PSMA-11, respectively, in not only kidneys and salivary glands but also in prostate cancer lesions 30-31. This suggests that there might be species differences for the distribution of PSMA and off-targets in prostate tumors, kidneys and salivary glands. Further clinical trials of ^68^Ga- and ^177^Lu-labeled HTK03149 are needed to confirm if the observation in the mouse model can be reproduced with prostate cancer patients.

## Conclusions

Replacing Glu in the PSMA-targeting Lys-urea-Glu pharmacophore of the previously reported [^68^Ga]Ga-HTK03041 with Asp and Aad generated [^68^Ga]Ga-HTK03161 and [^68^Ga]Ga-HTK03149, respectively. Both [^68^Ga]Ga-HTK03161 and [^68^Ga]Ga-HTK03149 retain excellent PSMA-targeting capability but show minimal uptake in normal organs/tissues including kidneys and salivary glands. Lys-urea-Asp and Lys-urea-Aad are, therefore, promising for the design of PSMA-targeting radioligands especially for radiotherapeutic agents to minimize toxicity to kidneys and salivary glands.

## Supplemental Material

Detailed procedures and results for the synthesis and purification of DOTA-conjugated PSMA-targeting ligands (Table S1), their nonradioactive Ga- and Lu-complexed standards (Table S2), and ^68^Ga- and ^177^Lu-labeled analogs (Table S3), biodistribution data (Tables S4-S6), the procedures and results for radiation dosimetry calculations (Tables S7-S8), and the change of tumor volume and mouse body weight for the radioligand therapy studies (Figures S1-S8) are provided in the Supplemental Information.

## Supplementary Material

Supplementary methods, figures and tables.

## Figures and Tables

**Table 1:** Summary of the radioligand therapy study results of [^177^Lu]Lu-HTK03149.

Group	Treatment (n = 7 or 8)	Injected radioactivity (MBq)		Tumor volume (mm^3^)	Median survival (days)
		Theoretical	Measured (mean±SD)	Day 0 (mean±SD)	
A	PBS	-	-	187±44	23
B	^nat^Lu-HTK03149	-	-	177±33	18
C	[^177^Lu]Lu-HTK03149	9.3	9.3±0.3	195±55	52
D	[^177^Lu]Lu-HTK03149	18.5	18.2±0.3	177±55	63
E	[^177^Lu]Lu-HTK03149	37	37.6±0.6	186±48	88
F	[^177^Lu]Lu-HTK03149	74	73.1±1.5	175±92	142
G	[^177^Lu]Lu-HTK03149	148	140.4±4.8	127±103	>180
H	[^177^Lu]Lu-PSMA-617	37	37.8±1.7	209±112	63

## Abbreviations

Aad: 2-aminoadipic acid
Api: 2-aminopimelic acid
Asp: aspartic acid
ATCC: American Type Culture Collection
CT: computed tomography
DOTA: 1,4,7,10-tetraazacyclododecane-1,4,7,10-tetraacetic acid
FDA: Food and Drug Administration
Glu: glutamic acid
HPLC: high performance liquid chromatography
PBS: phosphate-buffered saline
PET: positron emission tomography
PMPA: 2-(phosphonomethyl)pentanedioic acid
PSMA: prostate-specific membrane antigen
RPMI: Roswell Park Memorial Institute
SPECT: single-photon emission computed tomography
%IA/g: percent injected activity per gram of tissue
