# Supplementary material for: What a difference a methylene makes: replacing Glu with Asp or Aad in the Lys-urea-Glu pharmacophore of PSMA-targeting radioligands to reduce kidney and salivary gland uptake
Source: Theranostics. 2022 Aug 21;12(14):6179–88. doi: 10.7150/thno.76571 (PMC9475448; doi:10.7150/thno.76571)
Supplement: Supplementary file 1 — Supplementary methods, figures and tables. [file thnov12p6179s1.pdf]

SUPPLEMENTAL INFORMATION

General methods

All chemicals and solvents were obtained from commercial sources and used without further purification. [^{177}Lu]Lu-PSMA-617 was prepared following previously published procedures [1]. PSMA-targeting peptides were synthesized using the solid phase approach on an AAPPTec (Louisville, KY) Endeavor 90 peptide synthesizer. Purification and quality control of nonradioactive and radiolabeled peptides were performed using one of the Agilent (Santa Clara, CA) HPLC systems: (A) a model 1260 Infinity II preparative binary pump, a model 1260 Infinity variable wavelength detector (set at 220 nm), and a 1290 Infinity II preparative open-bed fraction collector; (B) a model 1260 Infinity quaternary pump and a model 1200 variable wavelength detector (set at 220 nm); and (C) a model 1200 quaternary pump, a model 1200 UV absorbance detector (set at 220 nm), and a Bioscan (Washington, DC) NaI scintillation detector. The HPLC columns used were (A) a preparative column (Gemini, NX-C18, 5 μ, 50 × 30 mm); (B) a semi-preparative column (Luna C18, 5 μ, 250 × 10 mm); and (C) an analytical column (Luna C18, 5 μ, 250 × 4.6 mm) purchased from Phenomenex (Torrance, CA). The collected HPLC eluates containing the desired peptide were lyophilized using a Labconco (Kansas City, MO) FreeZone 4.5 Plus freeze-drier. Mass analyses were performed using an AB SCIEX (Framingham, MA) 4000 QTRAP mass spectrometer system with an ESI ion source. C18 Sep-Pak cartridges (1 cm^3, 50 mg) were obtained from Waters (Milford, MA). ^{68}Ga was eluted from an iThemba Labs (Somerset West, South Africa) generator, and purified according to the previously published procedures using a DGA resin column from Eichrom Technologies LLC (Lisle, IL) [2]. $^{177}LuCl_3$ solution was ordered from ITG Isotope Technologies Garching Gmbh (Garching, Germany). Radioactivity of ^{68}Ga- and ^{177}Lu-labeled peptides was measured using a Capintec (Ramsey, NJ) CRC®-25R/W dose calibrator, and the radioactivity of mouse tissues collected from biodistribution studies were counted using a Perkin Elmer (Waltham, MA) Wizard2 2480 automatic gamma counter.

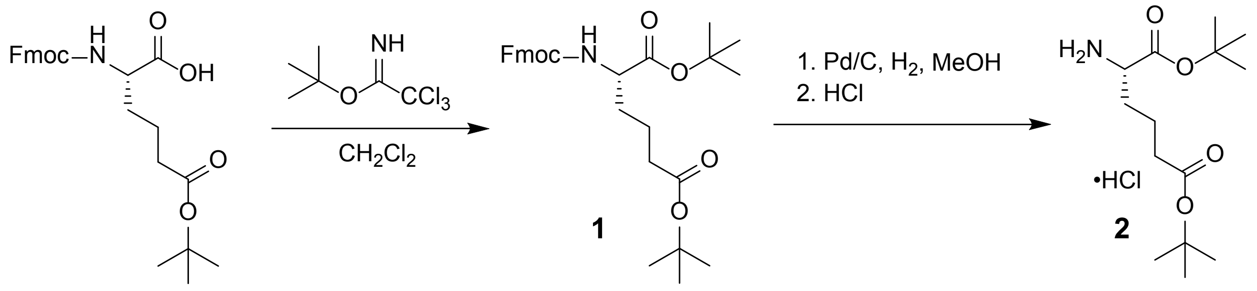

Scheme 1: Synthesis of L-Aad(OtBu)-OtBu.HCl (**2**)

Synthesis of Fmoc-L-Aad(OtBu)-OtBu (1)

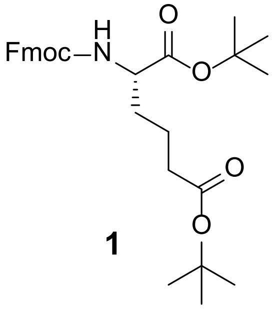

1

Fmoc-L-Aad(OtBu)-OH (2.20 g, 5 mmol) in CH_2Cl_2 (20 mL) was added *tert*-butyl 2,2,2-trichloroacetimidate (2.18 g, 10 mmol). The resulting mixture was stirred at room temperature for 18 h, and then purified by flash column chromatography eluted with 1:2 diethyl ether/hexanes to obtain **1** (2.20 g, 89%) as a colorless thick oil. ^{1}H NMR (400 MHz, $CDCl_3$) δ 7.85 (Ar-*H*, d, *J* = 7.5 Hz, 2H), 7.70 (Ar-*H*, d, *J* = 7.4 Hz, 2H), 7.49 (Ar-*H*, t, *J* = 7.4 Hz, 2H), 7.41 (Ar-*H*, td, *J* = 7.4, 1.1 Hz, 2H), 5.50 (N*H*, d, *J* = 8.0 Hz, 1H), 4.47 (OCH_2, d, *J* = 7.2 Hz, 2H), 4.40 – 4.34 (OCH_2CH, m, 1H), 4.34 – 4.29 (CH_2CH, m, 1H), 2.35 (COCH_2, t, *J* = 6.8 Hz, 2H), 1.87 – 1.67 (CH_2CH_2, m, 4H), 1.57 (*t-Bu*, s, 9H), 1.54 (*t-Bu*, s, 9H). MS (ESI) calculated for $C_{29}H_{37}NO_6$ 495.3, found $[M + H]^+$ 496.5.

Synthesis of L-Aad(OtBu)-OtBu.HCl (2)

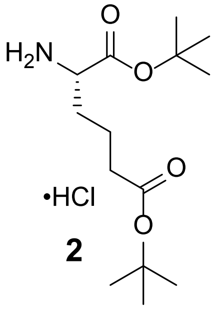

2

Compound **1** (2.13 g, 4.3 mmol) in MeOH (50 mL) was added palladium on carbon (10 wt. %, 300 mg). The resulting solution was hydrogenated using a balloon for 21 h. The mixture was filtered through celite, and concentrated under reduced pressure. The residue was dissolved in diethyl ether (150 mL), and the resulting solution was added HCl (4.0 M in 1,4-dioxane, 3 mL). After stirring for 5 min, the resulting precipitate was collected by filtration and dried under reduced pressure to yield **2** (1.26 g, 95%) as a white powder. ^{1}H NMR (400 MHz, $CDCl_3$) δ 8.87 (NH_3, d, *J* = 2.3 Hz, 3H), 4.15 – 3.94 (C*H*, m, 1H), 2.38 (COCH_2, t, *J* = 7.1 Hz, 2H), 2.13 (CHCH_2, dd, *J* = 14.9, 7.6 Hz, 2H), 2.03 – 1.77 (CH_2CH_2, m, 2H), 1.59 (*t-Bu*, s, 9H), 1.51 (*t-Bu*, s, 9H). MS (ESI) calculated for $C_{14}H_{27}NO_4$ 273.2, found $[M + H]^+$ 274.5.

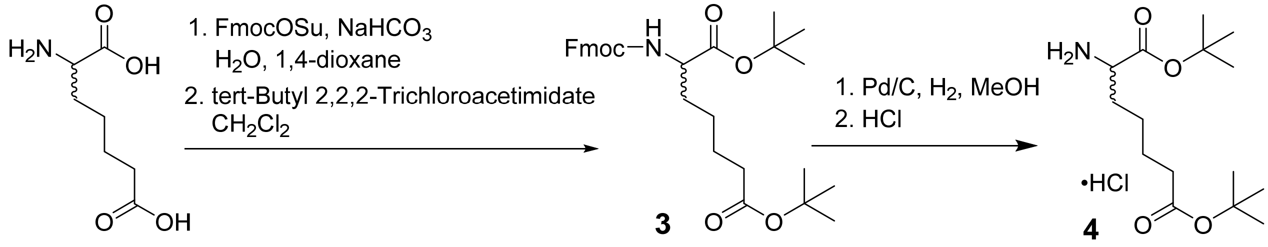

Scheme 2: Synthesis of Api(OtBu)-OtBu.HCl (**4**)

Synthesis of Fmoc-Api(OtBu)-OtBu (**3**)

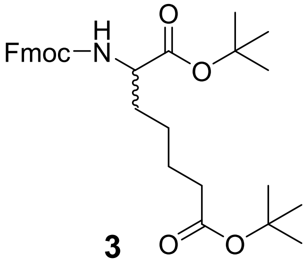

2-Aminopimelic acid (Api, 1.75 g, 10 mmol) and $NaHCO_3$ (2.1 g, 25 mmol) in water (40 mL) was added FmocOSu (3.37g, 10 mmol) in 1,4-dioxane (40 mL). The resulting solution was stirred at room temperature for 21 h. After washing with diethyl ether (60 mL × 2), the aqueous phase was acidified with concentrated HCl to pH 2. The acidic solution was extracted with ethyl acetate (100 mL × 2), and the organic phases were combined, dried over anhydrous $MgSO_4$, and evaporated to give 4.37 g crude Fmoc-protected 2-aminopimelic acid as a white solid.

The crude Fmoc-protected 2-aminopimelic acid (4.37 g) in CH_2Cl_2 (30 mL) was added *tert*-butyl 2,2,2-trichloroacetimidate (8.74 g, 40 mmol). The resulting mixture was stirred at room temperature for 22 h, and then purified by flash column chromatography eluted with 1:2 diethyl ether/hexanes to obtain **3** (2.53 g, 50%) as a colorless thick oil. ^{1}H NMR (400 MHz, $CDCl_3$) δ 7.76 (Ar-*H*, d, *J* = 7.5 Hz, 2H), 7.66 (Ar-*H*, d, *J* = 4.8 Hz, 2H), 7.51 (Ar-*H*, dt, *J* = 7.3, 4.1 Hz, 2H), 7.40 (Ar-*H*, dd, *J* = 9.0, 6.0 Hz, 2H), 5.32 (N*H*, d, *J* = 8.1 Hz, 1H), 4.38 (OCH_2, d, *J* = 7.1 Hz, 2H), 4.27 (OCH_2C*H*, t, *J* = 7.3 Hz, 1H), 4.22 (CH_2C*H*, t, *J* = 7.1 Hz, 1H), 2.23 – 2.20 (COCH_2, m, 2H), 1.70 – 1.68 (CHCH_2, m, 2H), 1.63 – 1.58 (CH_2CH_2, m, 4H), 1.44 (*t-Bu*, s, 18H). MS (ESI) calculated for $C_{30}H_{39}NO_6$ 509.3, found $[M + Na]^+$ 532.3.

Synthesis of Api(OtBu)-OtBu.HCl (4)

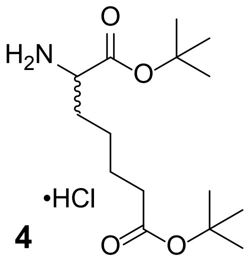

•HCl

4

Compound **3** (2.49 g, 4.9 mmol) in MeOH (80 mL) was added palladium on carbon (10 wt. %, 500 mg). The resulting solution was hydrogenated using a balloon for 16 h. The mixture was filtered through celite, and concentrated under reduced pressure. The residue was dissolved in diethyl ether (150 mL), and the resulting solution was added HCl (4.0 M in 1,4-dioxane, 3 mL). After stirring for 5 min, the resulting precipitate was collected by filtration and dried under reduced pressure to yield **4** (1.35 g, 85%) as a white powder. ^{1}H NMR (400 MHz, D_2O) δ 4.01 (C*H*, t, *J* = 6.3 Hz, 1H), 2.34 (COCH_2, t t, *J* = 7.2 Hz, 2H), 1.99 – 1.89 (CHCH_2, m, 2H), 1.64 (CH_2CH_2, m, 3H), 1.52 (*t-Bu*, s, 9H), 1.46 (*t-Bu*, s, 9H), 1.45 – 1.33 (CH_2CH_2, m, 2H). MS (ESI) calculated for $C_{15}H_{29}NO_4$ 287.2, found $[M + H]^+$ 288.2.

Synthesis of DOTA-conjugated peptidomimetics

The peptidomimetic PSMA-targeting Asp- (for HTK03161), Aad- (For HTK03149), and racemic Api- (for HTK03189A and HTK03189B) ureido-lysine moieties were synthesized by solid-phase peptide chemistry. Fmoc-Lys(ivDde)-Wang resin (0.1 mmol, 0.58 mmol/g loading) was suspended in DMF for 30 min. Fmoc was then removed by treating the resin with 20% piperidine in DMF (3 × 8 min). To generate the isocyanate derivative, a solution of L-aspartic acid di-tert-butyl ester hydrochloride (140.9 mg, 0.5 mmol, 5 eq relative to resin), L-2-aminoadipic acid (Aad) di-tert-butyl ester hydrochloride (154.9 mg, 0.5 mmol, 5 eq relative to resin), or 2-aminopimelic acid (Api) di-tert-butyl ester hydrochloride (161.9 mg, 0.5 mmol, 5 eq relative to resin) and DIEA (287.4 µL, 1.65 mmol) in CH_2Cl_2 (5 mL) was cooled to −78 °C in a dry ice/acetone bath. Triphosgene (49.0 mg, 0.165 mmol) was dissolved in CH_2Cl_2 (5 mL), and the resulting solution was added dropwise to the reaction at −78 °C. The reaction was then allowed to warm to room temperature and stirred for 30 minutes to give a solution of the isocyanate derivative. After which another 87.1 µL DIEA (0.5 mmole) was added, and then added to the lysine-immobilized resin and reacted for 16 h. After washing the resin with DMF, the ivDde-protecting group was removed with 2% hydrazine in DMF (5 × 5 min). Fmoc-Ala(9-Anth)-OH (4 eq.) was then coupled to the side chain of Lys using HATU (4 eq.), and DIEA (7 eq.). Afterwards, elongation was continued with the addition of Fmoc-tranexamic acid, and finally DOTA-tris(*t*-butyl)ester.

The peptides were deprotected and simultaneously cleaved from the resin by treating with 95/5 trifluoroacetic acid (TFA)/triisopropylsilane for 2 h at room temperature. After filtration, the peptides were precipitated by the addition of cold diethyl ether to the TFA solution. The crude peptides were purified by HPLC. The eluates containing the desired peptides were collected, pooled, and lyophilized. The HPLC conditions, retention times, isolated yields and MS confirmations of these DOTA-conjugated peptidomimetics are provided in Table S1.

Synthesis of nonradioactive Ga-complexed standards

To prepare nonradioactive Ga-complexed standards, a solution of the DOTA-conjugated precursor was incubated with $GaCl_3$ (5 eq.) in NaOAc buffer (0.1 M, 500 µL, pH 4.2) at 80 °C

for 15 min. The reaction mixture was then purified by HPLC, and the HPLC eluates containing the desired peptide were collected, pooled, and lyophilized. The HPLC conditions, retention times, isolated yields and MS confirmations of these nonradioactive Ga-complexed standards are provided in Table S2.

Synthesis of Lu-HTK03149

To prepare the nonradioactive Lu-complexed standard, a solution of HTK03149 was incubated with $LuCl_3$ (5 eq.) in NaOAc buffer (0.1 M, 500 µL, pH 4.2) at 95 °C for 15 min. The reaction mixture was then purified by HPLC, and the HPLC eluates containing the desired peptide were collected, pooled, and lyophilized. The HPLC condition, retention time, isolated yield and MS confirmations of nonradioactive Lu-HTK03149 are provided in Table S2.

Synthesis of ^{68}Ga-labeled compounds

The radiolabeling experiments were performed following previously published procedures (*2*). Purified $^{68}GaCl_3$ in 0.5 mL water was added into a 4-mL glass vial preloaded with 0.7 mL of HEPES buffer (2 M, pH 5.0) and 50 µg precursor. The radiolabeling reaction was carried out under microwave heating for 1 min. The reaction mixture was purified by HPLC using the semi-preparative column. The eluate fraction containing the radiolabeled product was collected, diluted with water (50 mL), and passed through a C18 Sep-Pak cartridge that was pre-washed with ethanol (10 mL) and water (10 mL). After washing the C18 Sep-Pak cartridge with water (10 mL), the ^{68}Ga-labeled product was eluted off the cartridge with ethanol (0.4 mL), and diluted with saline for imaging and biodistribution. Quality control was performed using the analytical column. The HPLC conditions and retention times are provided in Table S3. These ^{68}Ga-labeled PSMA-targeting ligandligands were obtained in 63-81% decay-corrected radiochemical yields with > 52 GBq/µmol molar activity and ≥ 95% radiochemical purity.

Synthesis of [^{177}Lu]Lu-HTK03149

[^{177}Lu]$LuCl_3$ (638–988 MBq in 10–20 µL) was added to a solution of HTK03149 (25 µg) in NaOAc buffer (0.5 mL, 0.1 M, pH 4.5). The mixture was incubated at 90 °C for 15 min, and then purified by HPLC using the semi-preparative column. The eluate fraction containing the radiolabeled product was collected, diluted with water (50 mL), and passed through a C18 Sep-Pak cartridge that was pre-washed with ethanol (10 mL) and water (10 mL). After washing the C18 Sep-Pak cartridge with water (10 mL), the ^{177}Lu-labeled product was eluted off the cartridge with ethanol (0.4 mL), and diluted with saline for imaging and biodistribution. Quality control was performed using the analytical column. The HPLC conditions and retention times are provided in Table S3. [^{177}Lu]Lu-HTK03149 was obtained in 59-65% decay-corrected radiochemical yields with 146-192 GBq/µmol molar activity and > 96% radiochemical purity.

Radiation dosimetry calculation

Internal dosimetry estimates were calculated using the organ level internal dose assessment (OLINDA) software v.2.2. These estimates were performed for the mouse using the 25g MOBY phantom [3], and for the tumors using the previously reported unit density sphere model [4]. Both the phantom and the sphere model are available in OLINDA and require the input of the total number of decays normalized by injected activity in units of MBq×h/MBq for each of the source organ/tumor.

The biodistribution data (available in the Supplemental Tables 5-6) were used to determine the kinetics input values required by OLINDA. First, each of the values was decayed to its corresponding time point (the values on the table are shown at injection time). Then the different time-points of the uptake data (%IA/g) for each organ were fitted to both mono-exponential ($\frac{\%ID}{g} = ae^{-bt}$) and bi-exponential ($\frac{\%ID}{g} = ae^{-bt} + ce^{-dt}$) functions using in-house software developed in Python. The best fit was selected based on maximizing the coefficient of determination (R^2) of the fit and minimizing the residuals. The areas under the curves were analytically calculated based on the parameters obtained from the best fit of each organ and this provided the kinetic input values required by OLINDA.

In the mouse case, the adrenals, blood, fat, and muscle are not modeled in the phantom. These organs were grouped together and included in what OLINDA calls the *remainder of the body*. Lastly, the numbers of decays in the tumors were also calculated based on the biodistribution data of the mice and the values were inputted into the sphere model available in OLINDA.

REFERENCES

1. Kuo H-T, Merkens H, Zhang Z, Uribe CF, Lau J, Zhang C, et al. Enhancing treatment efficacy of ^{177}Lu-PSMA-617 with the conjugation of an albumin-binding motif: preclinical dosimetry and endoradiotherapy studies. Mol Pharmaceutics. 2018; 15: 5183-91.

2. Lin K-S, Pan J, Amouroux G, Turashvili G, Mesak F, Hundal-Jabal N, et al. In vivo radioimaging of bradykinin receptor B1, a widely overexpressed molecule in human cancer. Cancer Res. 2015; 75: 387-93.

3. Keenan MA, Stabin MG, Segars WP, Fernald MJ. RADAR realistic animal model series for dose assessment. J Nucl Med. 2010; 51: 471–6.

4. Stabin MG, Konijnenberg MW. Re-evaluation of absorbed fractions for photons and electrons in spheres of various sizes. J Nucl Med. 2000; 41: 149–60.

Supplementary Table 1: HPLC purification conditions and MS characterizations of DOTA-conjugated PSMA-targeting ligands.

Compound name	HPLC conditions	Retention time (min)	Yield (%)	Calculated mass	Found
HTK03161	System A/Column A: 23% CH_3CN and 0.1% TFA in H_2O; flow rate 30 mL/min	7.9	17	$[M+H]^+$ 1078.5	$[M+H]^+$ 1078.5
HTK03149	System A/Column A: 24% CH_3CN and 0.1% TFA in H_2O; flow rate 30 mL/min	6.9	21	$[M+H]^+$ 1106.5	$[M+H]^+$ 1106.1
HTK03189A	System B/Column B: 28% CH_3CN and 0.1% TFA in H_2O; flow rate 4.5 mL/min	13.9	20	$[M+H]^+$ 1120.6	$[M+H]^+$ 1120.6
HTK03189B	System B/Column B: 28% CH_3CN and 0.1% TFA in H_2O; flow rate 4.5 mL/min	15.5	16	$[M+H]^+$ 1120.6	$[M+H]^+$ 1120.5

Supplementary Table 2: HPLC purification conditions and MS characterizations of nonradioactive Ga- and Lu-complexed PSMA-targeting ligands.

Compound name	HPLC conditions	Retention time (min)	Yield (%)	Calculated mass	Found
Ga-HTK03161	System B/Column B: 29% CH_3CN and 0.1% TFA in H_2O; flow rate 30 mL/min	11.3	37	$[M+H]^+$ 1145.4	$[M+H]^+$ 1146.1
Ga-HTK03149	System A/Column A: 24% CH_3CN and 0.1% TFA in H_2O; flow rate 30 mL/min	10.9	90	$[M+H]^+$ 1173.5	$[M+H]^+$ 1173.5
Lu-HTK03149	System B/Column B: 29% CH_3CN and 0.1% TFA in H_2O; flow rate 4.5 mL/min	8.4	91	$[M+H]^+$ 1278.5	$[M+H]^+$ 1278.4
Ga-HTK03189A	System B/Column B: 30% CH_3CN and 0.1% TFA in H_2O; flow rate 4.5 mL/min	13.0	53	$[M+H]^+$ 1186.5	$[M+H]^+$ 1186.4
Ga-HTK03189B	System B/Column B: 30% CH_3CN and 0.1% TFA in H_2O; flow rate 4.5 mL/min	13.9	42	$[M+H]^+$ 1186.5	$[M+H]^+$ 1186.3

Supplementary Table 3: HPLC conditions for the purification and quality control of ^{68}Ga- and ^{177}Lu-labeled PSMA-targeting ligands.

Compound name	HPLC conditions		Retention time (min)
[^{68}Ga]Ga-HTK03161	Semi-prep	27% CH_3CN and 0.1% TFA in H_2O; flow rate 4.5 mL/min	21.4
	QC	29% CH_3CN and 0.1% TFA in H_2O; flow rate 2.0 mL/min	6.6
[^{68}Ga]Ga-HTK03149	Semi-prep	28% CH_3CN and 0.1% TFA in H_2O; flow rate 4.5 mL/min	19.6
	QC	28% CH_3CN and 0.1% TFA in H_2O; flow rate 2 mL/min	9.7
[^{68}Ga]Ga-HTK03189A	Semi-prep	29% CH_3CN and 0.1% TFA in H_2O; flow rate 4.5 mL/min	18.9
	QC	32% CH_3CN and 0.1% TFA in H_2O; flow rate 2.0 mL/min	5.7
[^{68}Ga]Ga-HTK03189B	Semi-prep	29% CH_3CN and 0.1% TFA in H_2O; flow rate 4.5 mL/min	20.6
	QC	32% CH_3CN and 0.1% TFA in H_2O; flow rate 2.0 mL/min	6.0
[^{177}Lu]Lu-HTK03149	Semi-prep	27% CH_3CN and 0.1% TFA in H_2O; flow rate 4.5 mL/min	19.7
	QC	29% CH_3CN and 0.1% TFA in H_2O; flow rate 2.0 mL/min	6.0

Supplementary Table 4: Biodistribution (mean ± SD) and uptake ratios of ^{68}Ga-labeled PSMA-targeting ligandligands in LNCaP tumor-bearing mice. The mice in the blocked group were co-injected with DCFPyL (0.5 mg). Statistical analysis was conducted for the data of [^{68}Ga]Ga-HTK03149 with/without co-injection of DCFPyL (unpaired, one-tailed *t*-test: *, $p < 0.05$; **, $p < 0.01$; ***, $p < 0.001$).

Tissue (%IA/g)	**[^{68}Ga]Ga-HTK03161**	**[^{68}Ga]Ga - HTK03149**		**[^{68}Ga]Ga - HTK03189A**	**[^{68}Ga]Ga - HTK03189B**
	1 h (n = 4)	1 h (n = 6)	1 h blocked (n = 4)	1 h (n = 5)	1 h (n = 5)
Blood	1.13 ± 0.19	0.70 ± 0.17	0.67 ± 0.04	0.89 ± 0.22	1.06 ± 0.25
Fat	0.15 ± 0.04	0.17 ± 0.15	0.08 ± 0.01	0.18 ± 0.11	0.17 ± 0.10
Testes	0.54 ± 0.51	0.23 ± 0.13	0.19 ± 0.02	0.23 ± 0.11	0.26 ± 0.05
Intestines	0.31 ± 0.09	0.24 ± 0.05	0.21 ± 0.04	0.31 ± 0.18	0.28 ± 0.07
Stomach	0.10 ± 0.04	0.07 ± 0.01	0.04 ± 0.00***	0.11 ± 0.06	0.11 ± 0.03
Spleen	0.27 ± 0.06	0.27 ± 0.05	0.15 ± 0.04**	0.21 ± 0.10	0.20 ± 0.05
Liver	0.28 ± 0.05	0.25 ± 0.06	0.22 ± 0.03	0.34 ± 0.15	0.39 ± 0.08
Pancreas	0.18 ± 0.03	0.13 ± 0.02	0.12 ± 0.01	0.15 ± 0.04	0.18 ± 0.05
Adrenal glands	0.41 ± 0.07	0.33 ± 0.11	0.22 ± 0.03*	0.35 ± 0.06	0.37 ± 0.15
Kidneys	4.41 ± 1.26	4.15 ± 1.46	1.93 ± 0.39**	2.65 ± 0.69	2.13 ± 0.45
Lungs	0.75 ± 0.13	0.53 ± 0.09	0.49 ± 0.03	0.68 ± 0.12	0.82 ± 0.22
Heart	0.32 ± 0.05	0.21 ± 0.03	0.19 ± 0.01	0.24 ± 0.06	0.30 ± 0.06
Tumor	12.7 ± 1.91	19.1 ± 6.37	0.77 ± 0.06***	2.10 ± 0.28	0.67 ± 0.15
Muscle	0.15 ± 0.04	0.11 ± 0.04	0.08 ± 0.01	0.14 ± 0.04	0.12 ± 0.03
Bone	0.11 ± 0.02	0.11 ± 0.04	0.07 ± 0.01	0.12 ± 0.03	0.10 ± 0.02
Brain	0.02 ± 0.00	0.02 ± 0.00	0.02 ± 0.00	0.02 ± 0.00	0.03 ± 0.00
Thyroid	0.29 ± 0.05	0.20 ± 0.05	0.18 ± 0.01	0.25 ± 0.05	0.29 ± 0.08
Salivary glands	0.23 ± 0.05	0.22 ± 0.06	0.14 ± 0.01*	0.20 ± 0.04	0.22 ± 0.04
Lacrimal glands	0.12 ± 0.06	0.15 ± 0.09	0.14 ± 0.08	0.18 ± 0.18	0.13 ± 0.04
Tumor:Blood	11.4 ± 1.73	29.5 ± 15.8	1.16 ± 0.13**	2.41 ± 0.29	0.65 ± 0.15
Tumor:Muscle	89.4 ± 36.1	185 ± 79.6	7.18 ± 3.58**	16.5 ± 4.08	5.68 ± 1.12
Tumor:Kidney	3.03 ± 0.84	5.44 ± 3.88	0.41 ± 0.09*	0.83 ± 0.21	0.32 ± 0.05
Tumor:Salivary gland	57.2 ± 7.11	97.3 ± 59.2	5.53 ± 0.39**	10.5 ± 1.07	3.08 ± 0.53
Blood:Salivary gland	5.08 ± 0.62	3.21 ± 0.64	4.80 ± 0.54**	4.39 ± 0.44	4.86 ± 1.16

Supplementary Table 5: Biodistribution (mean ± SD) and uptake ratios of [^{177}Lu]Lu-PSMA-617 in LNCaP tumor-bearing mice.

Tissue (%IA/g)	1 h (n = 8)	4 h (n = 8)	24 h (n = 8)	72 h (n = 8)	120 h (n = 8)
Blood	0.750 ± 0.119	0.031 ± 0.017	0.004 ± 0.001	0.002 ± 0.001	0.002 ± 0.001
Fat	0.327 ± 0.069	0.028 ± 0.011	0.009 ± 0.008	0.006 ± 0.012	0.002 ± 0.001
Testes	0.433 ± 0.068	0.055 ± 0.018	0.018 ± 0.003	0.014 ± 0.012	0.006 ± 0.002
Intestines	0.275 ± 0.032	0.135 ± 0.042	0.059 ± 0.039	0.062 ± 0.080	0.012 ± 0.011
Stomach	0.130 ± 0.054	0.029 ± 0.017	0.044 ± 0.011	0.096 ± 0.155	0.013 ± 0.013
Spleen	3.666 ± 1.342	0.222 ± 0.117	0.039 ± 0.007	0.016 ± 0.005	0.013 ± 0.004
Liver	0.224 ± 0.030	0.056 ± 0.012	0.043 ± 0.027	0.021 ± 0.004	0.016 ± 0.004
Pancreas	0.387 ± 0.074	0.031 ± 0.012	0.008 ± 0.002	0.003 ± 0.001	0.001 ± 0.001
Adrenal glands	3.025 ± 0.939	0.469 ± 0.527	0.032 ± 0.010	0.007 ± 0.004	0.005 ± 0.008
Kidneys	79.67 ± 11.68	13.06 ± 9.598	0.658 ± 0.159	0.181 ± 0.046	0.111 ± 0.054
Lungs	1.377 ± 0.152	0.123 ± 0.052	0.028 ± 0.005	0.011 ± 0.002	0.006 ± 0.001
Heart	0.294 ± 0.045	0.026 ± 0.006	0.009 ± 0.003	0.004 ± 0.001	0.002 ± 0.001
Tumor	16.48 ± 2.456	14.24 ± 1.804	13.18 ± 3.151	10.97 ± 2.122	12.25 ± 5.519
Muscle	0.177 ± 0.032	0.020 ± 0.004	0.005 ± 0.001	0.004 ± 0.004	0.002 ± 0.002
Bone	0.118 ± 0.026	0.017 ± 0.007	0.009 ± 0.005	0.006 ± 0.003	0.003 ± 0.004
Brain	0.028 ± 0.007	0.011 ± 0.001	0.010 ± 0.002	0.008 ± 0.002	0.007 ± 0.001
Thyroid	0.556 ± 0.073	0.065 ± 0.027	0.0121 ± 0.120	0.009 ± 0.002	0.005 ± 0.001
Salivary glands	1.775 ± 0.381	0.142 ± 0.039	0.018 ± 0.003	0.012 ± 0.003	0.005 ± 0.002
Lacrimal glands	0.068 ± 0.022	0.079 ± 0.056	0.050 ± 0.024	0.023 ± 0.009	0.021 ± 0.010
Tumor:Blood	22.5 ± 4.07	593 ± 338	4045 ± 1693	5784 ± 1756	8895 ± 4881
Tumor:Muscle	94.3 ± 12.8	748 ± 180	2905 ± 453	4712 ± 3587	11627 ± 7865
Tumor:Kidney	0.21 ± 0.04	1.58 ± 0.98	21.6 ± 8.83	62.4 ± 11.1	126 ± 76.0
Tumor:Salivary gland	9.81 ± 3.33	105 ± 28.0	371 ± 129	969 ± 241	3324 ± 2257
Blood:Salivary gland	0.44 ± 0.12	0.20 ± 0.06	0.10 ± 0.02	0.19 ± 0.08	0.42 ± 0.19

Supplementary Table 6: Biodistribution (mean ± SD) and uptake ratios of [^{177}Lu]Lu-HTK03149 in LNCaP tumor-bearing mice.

Tissue (%IA/g)	1 h (n = 4)	4 h (n = 4)	24 h (n = 4)	72 h (n = 4)	120 h (n = 4)
Blood	0.876 ± 0.073	0.167 ± 0.033	0.116 ± 0.007	0.067 ± 0.016	0.021 ± 0.006
Fat	0.136 ± 0.049	0.021 ± 0.003	0.017 ± 0.002	0.042 ± 0.042	0.014 ± 0.009
Testes	0.255 ± 0.019	0.056 ± 0.005	0.047 ± 0.003	0.053 ± 0.008	0.033 ± 0.012
Intestines	0.267 ± 0.063	0.185 ± 0.067	0.086 ± 0.047	0.088 ± 0.034	0.069 ± 0.046
Stomach	0.096 ± 0.020	0.049 ± 0.021	0.125 ± 0.112	0.140 ± 0.104	0.084 ± 0.041
Spleen	0.260 ± 0.045	0.089 ± 0.020	0.085 ± 0.012	0.142 ± 0.062	0.129 ± 0.079
Liver	0.264 ± 0.031	0.108 ± 0.010	0.103 ± 0.008	0.130 ± 0.034	0.079 ± 0.041
Pancreas	0.165 ± 0.028	0.034 ± 0.004	0.032 ± 0.005	0.033 ± 0.002	0.020 ± 0.007
Adrenal glands	0.352 ± 0.071	0.085 ± 0.007	0.073 ± 0.018	0.124 ± 0.052	0.024 ± 0.027
Kidneys	7.672 ± 1.346	1.666 ± 0.378	0.596 ± 0.109	0.679 ± 0.387	0.276 ± 0.064
Lungs	0.721 ± 0.056	0.169 ± 0.012	0.122 ± 0.015	0.108 ± 0.022	0.086 ± 0.076
Heart	0.281 ± 0.036	0.070 ± 0.013	0.065 ± 0.004	0.060 ± 0.014	0.034 ± 0.014
Tumor	13.96 ± 2.155	20.90 ± 2.988	13.80 ± 2.876	17.12 ± 4.702	16.39 ± 10.99
Muscle	0.182 ± 0.138	0.021 ± 0.005	0.017 ± 0.003	0.018 ± 0.003	0.012 ± 0.004
Bone	0.072 ± 0.020	0.016 ± 0.010	0.025 ± 0.003	0.026 ± 0.011	0.015 ± 0.007
Brain	0.022 ± 0.002	0.010 ± 0.001	0.006 ± 0.001	0.003 ± 0.002	0.002 ± 0.001
Thyroid	0.269 ± 0.021	0.066 ± 0.008	0.060 ± 0.002	0.079 ± 0.008	0.044 ± 0.015
Salivary glands	0.227 ± 0.062	0.051 ± 0.010	0.049 ± 0.007	0.051 ± 0.016	0.029 ± 0.007
Lacrimal glands	0.022 ± 0.006	0.029 ± 0.014	0.004 ± 0.005	0.006 ± 0.007	0.001 ± 0.002
Tumor:Blood	16.0 ± 2.44	12.6 ± 1.11	120 ± 31.1	258 ± 74.7	780 ± 451
Tumor:Muscle	101 ± 48.3	1028 ± 227	842 ± 216	985 ± 342	1336 ± 566
Tumor:Kidney	1.83 ± 0.21	12.9 ± 2.72	24.0 ± 7.26	28.1 ± 6.86	56.8 ± 26.2
Tumor:Salivary gland	63.4 ± 13.6	415 ± 60.5	286 ± 88.4	349 ± 109	533 ± 218
Blood:Salivary gland	3.98 ± 0.65	3.32 ± 0.59	2.37 ± 0.24	1.45 ± 0.69	0.75 ± 0.19

Supplementary Table 7: Radiation dose (mGy/MBq) of [^{177}Lu]Lu-HTK03149 and [^{177}Lu]Lu-PSMA-617 calculated from unit density sphere models for the LNCaP tumors.

Sphere/Tumor volume (mL)	[^{177}Lu]Lu-HTK03149	[^{177}Lu]Lu-PSMA-617
0.01	7.53E+04	3.07E+04
0.1	7.87E+03	3.21E+03
0.5	1.60E+03	6.51E+02
1	8.06E+02	3.28E+02
2	4.05E+02	1.65E+02
4	2.03E+02	8.28E+01
6	1.36E+02	5.53E+01
8	1.02E+02	4.15E+01
10	8.17E+01	3.33E+01
20	4.10E+01	1.67E+01
40	2.06E+01	8.38E+00
60	1.37E+01	5.60E+00
80	1.03E+01	4.21E+00
100	8.28E+00	3.37E+00
300	2.79E+00	1.13E+00
400	2.10E+00	8.54E-01
500	1.68E+00	6.85E-01
600	1.40E+00	5.72E-01
1000	8.48E-01	3.45E-01
2000	4.29E-01	1.75E-01
3000	2.88E-01	1.17E-01
4000	2.17E-01	8.86E-02
5000	1.75E-01	7.12E-02
6000	1.46E-01	5.96E-02

Supplementary Table 8: Radiation doses (mGy/MBq) of [^{177}Lu]Lu-HTK03149 and [^{177}Lu]Lu-PSMA-617 calculated for the major organs of 25-g mice using the OLINDA software.

Kinetics value [MBq-h/MBq]			Organ doses [mGy/MBq]		
Source organ	[^{177}Lu]Lu-HTK03149	[^{177}Lu]Lu-PSMA-617	Target organ	[^{177}Lu]Lu-HTK03149	[^{177}Lu]Lu-PSMA-617
Brain	2.23E-03	6.64E-03	Brain	2.35E+00	1.79E+00
Large intestine	9.24E-02	8.80E-03	Large intestine	1.59E+01	2.42E+00
Small intestine	2.76E-01	2.63E-02	Small intestine	1.57E+01	2.08E+00
Stomach	8.07E-03	2.34E-04	Stomach wall	1.57E+01	1.51E+00
Heart contents	1.94E-02	3.28E-03	Heart	9.38E+00	1.90E+00
Kidneys	2.41E-01	8.23E-01	Kidneys	6.72E+01	2.23E+02
Liver	3.91E-01	6.38E-02	Liver	2.09E+01	3.84E+00
Lungs	1.72E-02	4.85E-03	Lungs	1.62E+01	4.46E+00
Pancreas	1.79E-02	4.76E-03	Pancreas	7.72E+00	3.60E+00
Cortical bone	7.60E-02	8.28E-03	Skeleton	7.45E+00	1.25E+00
Spleen	2.30E-02	1.41E-02	Spleen	1.89E+01	1.15E+01
Testes	1.29E-02	4.23E-03	Testes	8.72E+00	3.04E+00
Thyroid	2.03E-03	4.01E-04	Thyroid	1.29E+01	2.78E+00
Urinary bladder contents	8.14E-01	8.04E-01	Urinary bladder wall	5.84E+02	5.76E+02
Remainder of the body	5.31E-01	1.68E-01	Remainder of the body	8.95E+00	6.89E+00

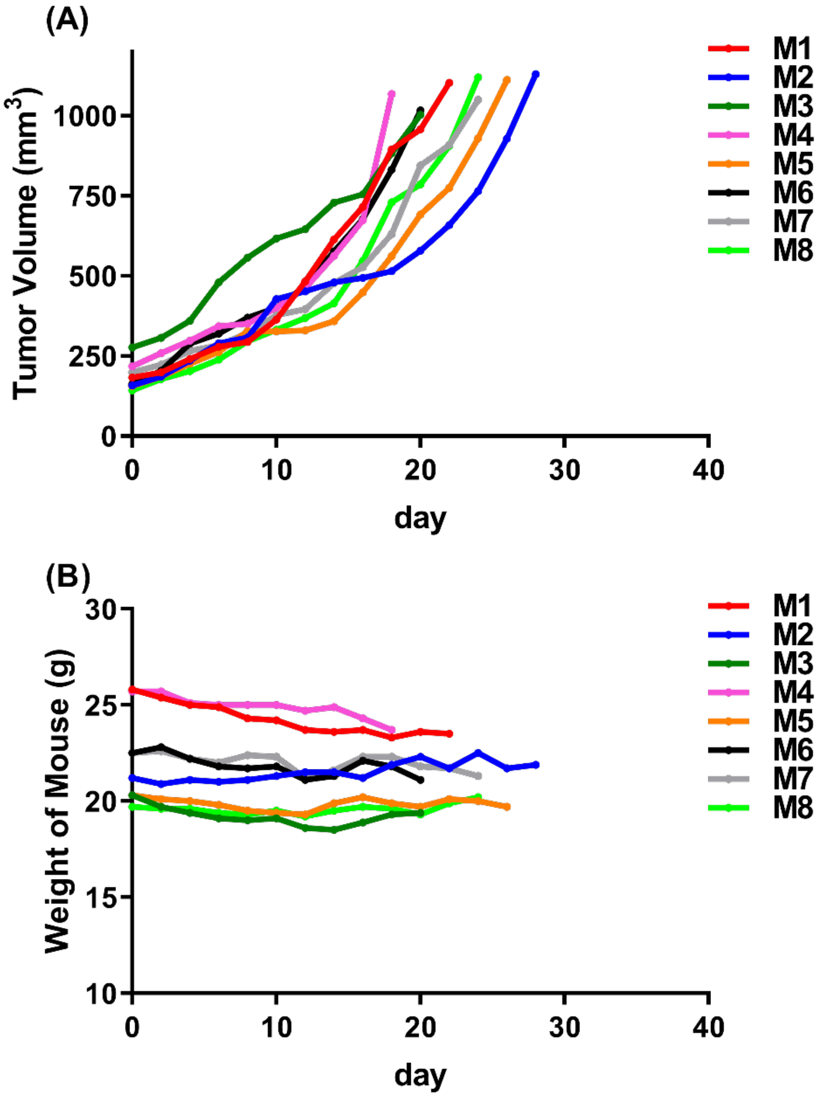

Supplementary Figure 1: Changes of (A) tumor volume and (B) body weight over time after mice were treated with PBS.

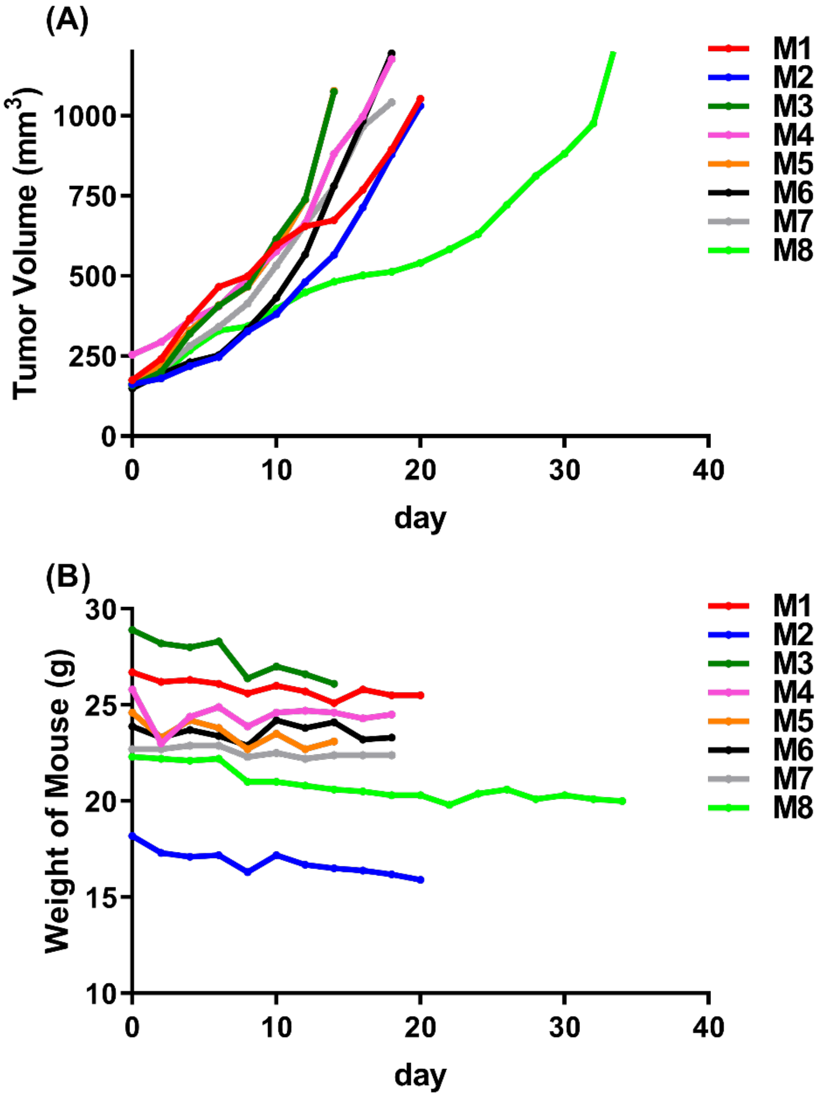

Supplementary Figure 2: Changes of (A) tumor volume and (B) body weight over time after mice were treated with ^{nat}Lu-HTK03149.

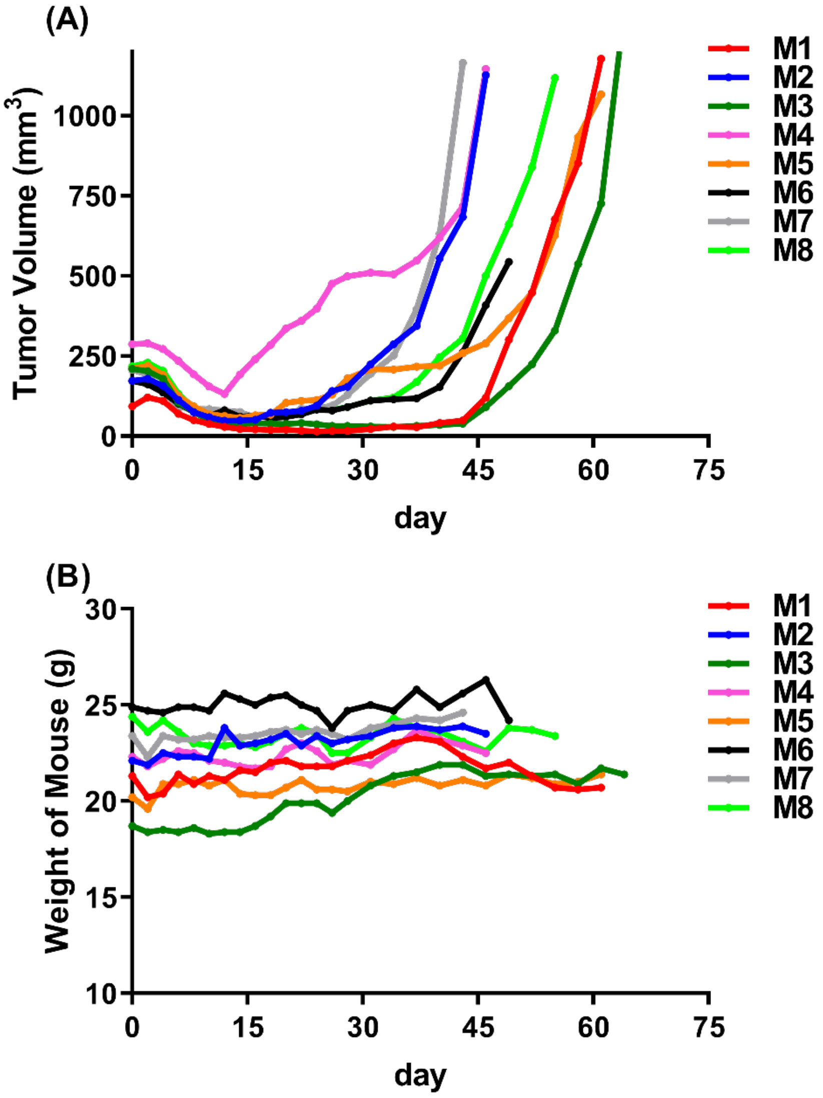

Supplementary Figure 3: Changes of (A) tumor volume and (B) body weight over time after mice were treated with [^{177}Lu]Lu-HTK03149 (9.3 MBq).

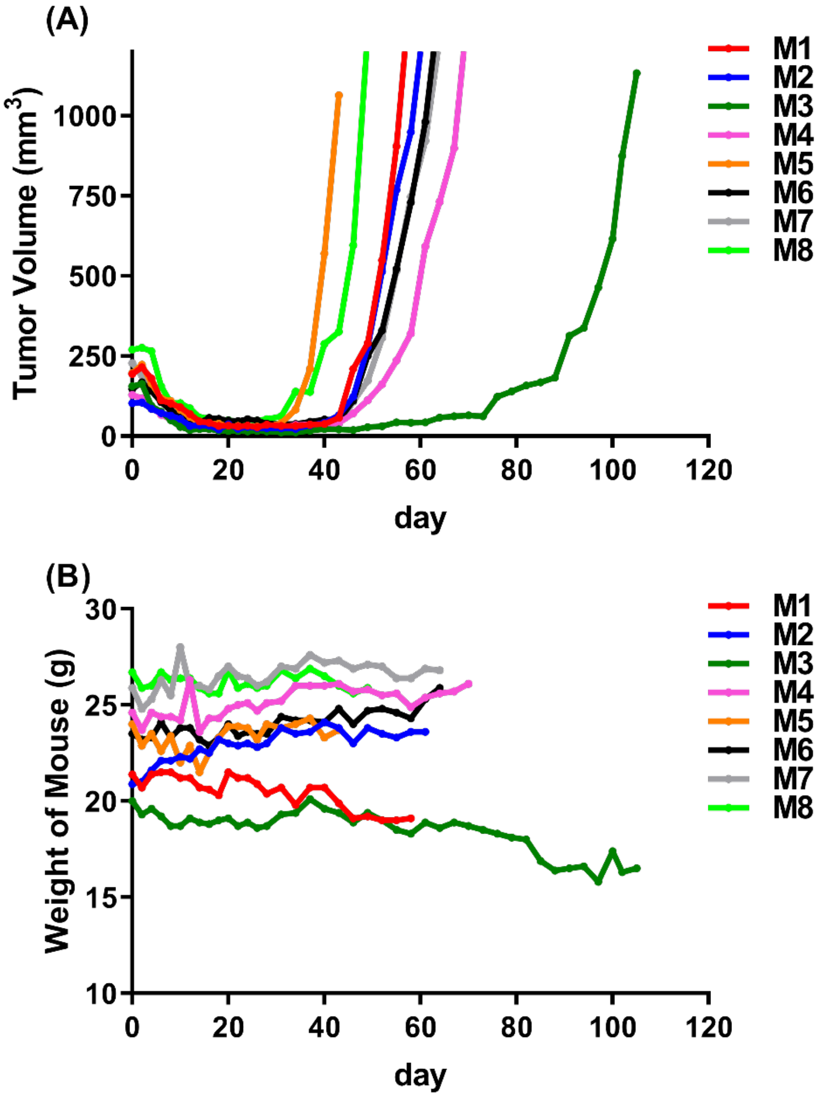

Supplementary Figure 4: Changes of (A) tumor volume and (B) body weight over time after mice were treated with [^{177}Lu]Lu-HTK03149 (18.5 MBq).

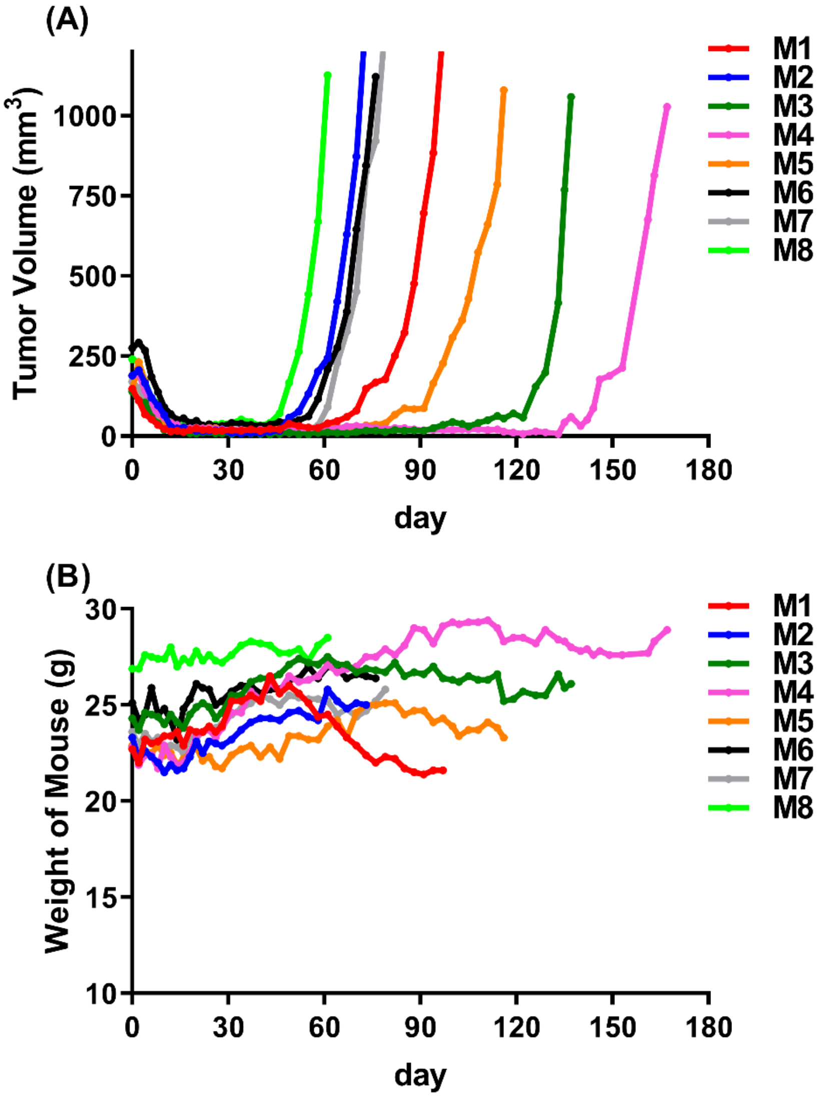

Supplementary Figure 5: Changes of (A) tumor volume and (B) body weight over time after mice were treated with [^{177}Lu]Lu-HTK03149 (37 MBq).

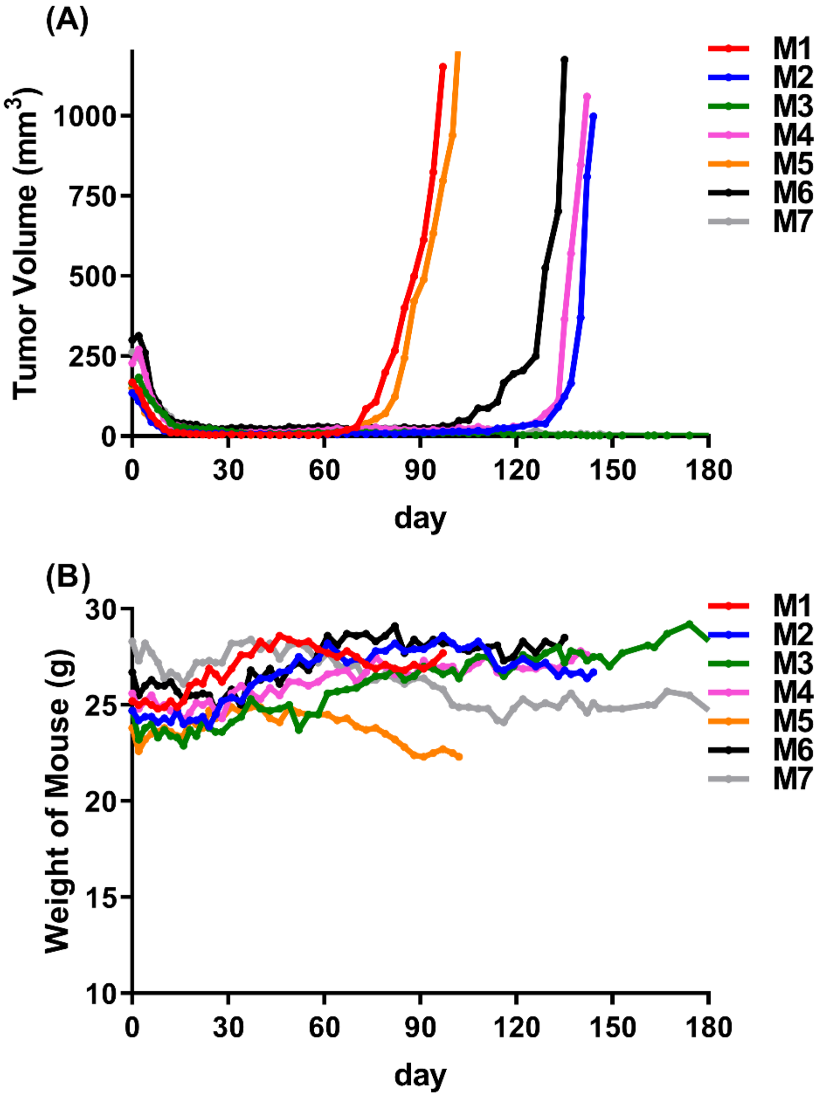

Supplementary Figure 6: Changes of (A) tumor volume and (B) body weight over time after mice were treated with [^{177}Lu]Lu-HTK03149 (74 MBq).

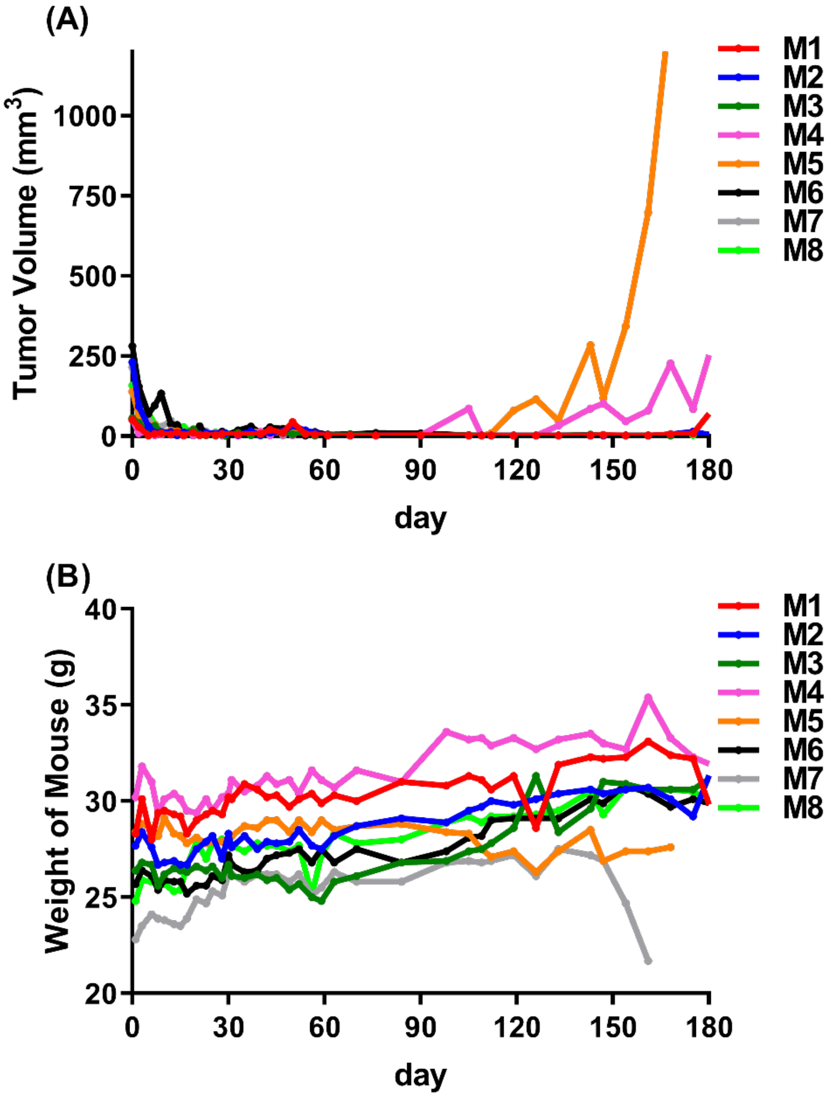

Supplementary Figure 7: Changes of (A) tumor volume and (B) body weight over time after mice were treated with [^{177}Lu]Lu-HTK03149 (148 MBq).

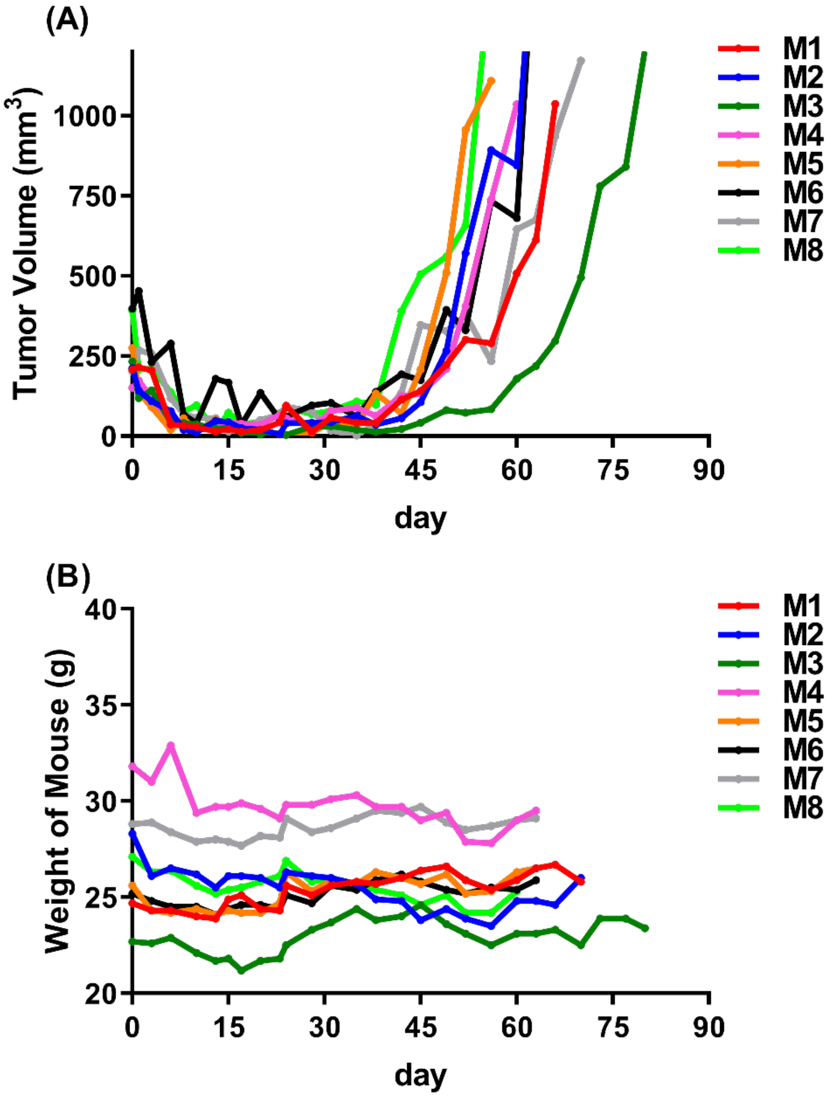

Supplementary Figure 8: Changes of (A) tumor volume and (B) body weight over time after mice were treated with [^{177}Lu]Lu-PSMA-617 (37 MBq).
